# Effectiveness of a Web-based and Mobile Therapy Chatbot on Anxiety and Depressive Symptoms in Subclinical Young Adults: Randomized Controlled Trial

**DOI:** 10.2196/47960

**Published:** 2024-03-20

**Authors:** Stanisław Karkosz, Robert Szymański, Katarzyna Sanna, Jarosław Michałowski

**Affiliations:** 1 Laboratory of Affective Neuroscience in Poznan SWPS University Warsaw Poland; 2 Center for Research on Personality Development in Poznan SWPS University Warsaw Poland

**Keywords:** chatbots, conversational agents, chatbot, conversational agent, artificial intelligence, mental health, depression, anxiety, depressive, cognitive distortions, young adults, randomized control trial, RCT, user experience, CBT, psychotherapy, cognitive behavioral therapy

## Abstract

**Background:**

There has been an increased need to provide specialized help for people with depressive and anxiety symptoms, particularly teenagers and young adults. There is evidence from a 2-week intervention that chatbots (eg, Woebot) are effective in reducing depression and anxiety, an effect that was not detected in the control group that was provided self-help materials. Although chatbots are a promising solution, there is limited scientific evidence for the efficacy of agent-guided cognitive behavioral therapy (CBT) outside the English language, especially for highly inflected languages.

**Objective:**

This study aimed to measure the efficacy of Fido, a therapy chatbot that uses the Polish language. It targets depressive and anxiety symptoms using CBT techniques. We hypothesized that participants using Fido would show a greater reduction in anxiety and depressive symptoms than the control group.

**Methods:**

We conducted a 2-arm, open-label, randomized controlled trial with 81 participants with subclinical depression or anxiety who were recruited via social media. Participants were divided into experimental (interacted with a fully automated Fido chatbot) and control (received a self-help book) groups. Both intervention methods addressed topics such as general psychoeducation and cognitive distortion identification and modification via Socratic questioning. The chatbot also featured suicidal ideation identification and redirection to suicide hotlines. We used self-assessment scales to measure primary outcomes, including the levels of depression, anxiety, worry tendencies, satisfaction with life, and loneliness at baseline, after the 2-week intervention and at the 1-month follow-up. We also controlled for secondary outcomes, including engagement and frequency of use.

**Results:**

There were no differences in anxiety and depressive symptoms between the groups at enrollment and baseline. After the intervention, depressive and anxiety symptoms were reduced in both groups (chatbot: n=36; control: n=38), which remained stable at the 1-month follow-up. Loneliness was not significantly different between the groups after the intervention, but an exploratory analysis showed a decline in loneliness among participants who used Fido more frequently. Both groups used their intervention technique with similar frequency; however, the control group spent more time (mean 117.57, SD 72.40 minutes) on the intervention than the Fido group (mean 79.44, SD 42.96 minutes).

**Conclusions:**

We did not replicate the findings from previous (eg, Woebot) studies, as both arms yielded therapeutic effects. However, such results are in line with other research of Internet interventions. Nevertheless, Fido provided sufficient help to reduce anxiety and depressive symptoms and decreased perceived loneliness among high-frequency users, which is one of the first pieces of evidence of chatbot efficacy with agents that use a highly inflected language. Further research is needed to determine the long-term, real-world effectiveness of Fido and its efficacy in a clinical sample.

**Trial Registration:**

ClinicalTrials.gov NCT05762939; https://clinicaltrials.gov/study/NCT05762939; Open Science Foundation Registry 2cqt3; https://osf.io/2cqt3

## Introduction

### Background

The interest in digital mental health apps has largely increased in recent years [1]. Their growing popularity results mainly from the pressure to use technology during the COVID-19 pandemic, which coincided with a fast deterioration in public mental health and the increasing quality of digital health technologies. Therapy applications have been proven to be helpful for clients not able to afford traditional therapy and for therapists seeking solutions to increase client engagement in therapy [2]. A recent meta-analysis [3] showed that using an internet-based intervention can be as effective as face-to-face therapy. Nevertheless, applications that are available on the market often lack appropriate scientific evidence of feasibility or efficacy [4].

One solution that seems to be especially promising is agent-guided cognitive behavioral therapy (AG-CBT [5]), in which interventions are provided by chatbots—applications backed by machine learning algorithms that mimic natural conversation while communicating with users via a chat interface [6]. The development of such applications has been approached in many different ways, but one bot that seems to be the most advanced so far is Woebot. Woebot is a self-help chatbot using CBT techniques such as psychoeducation, goal planning, and mood tracking to lower the levels of depression, anxiety, substance abuse, and, recently, postnatal depression [5,6].

In a randomized controlled trial (RCT), the use of Woebot for a period of 2 weeks has been proven to be more effective at reducing symptoms of anxiety and depression than the use of self-help materials prepared by the World Health Organization (WHO) [6]. Further studies have provided evidence that users can develop a bond with the Woebot on a similar level as the one that is built between the client and therapist during group CBT [5,7].

Previous experiences with English-speaking mental health care chatbots (eg. Woebot, Wysa, Youper [8-10]) have encouraged attempts to develop chatbots in other language versions, such as German [11], Chinese [12], Spanish [13], and Ukrainian [14], or even multilingual chatbots [15]. Currently, the development of such applications varies among high-income and low-income countries due to cross-cultural differences and specific obstacles [16]. In Poland, there is still a limited number of digital therapeutic solutions, although the need for them is growing. In the last few years, there has been a visible decline in mental health among Polish teenagers and young adults, which became especially severe during the COVID-19 pandemic [17].

To address that need, our team initiated the development of Fido—the first Polish therapy chatbot—that aims to provide mental health support to adolescents and young adults struggling with anxiety and depression. Our previous research on the interaction between humans and Fido showed that it is considerably user-friendly [18]. However, Fido still required an efficacy study, which is presented in this article along with the exploratory analysis of human–therapy chatbot interactions. We hope that this study will extend previous research in this field and enrich the discussion on agent-guided mental health treatment.

### Objectives

A previous study on human-chatbot interactions using Fido provided satisfactory results, suggesting that Fido is pleasant to use; however, it hasn’t been optimized yet and required further development in the area of user experience (UX) [18]. After its UX optimization, Fido has been ready to use in an efficacy study. Therefore, we performed the first RCT aimed at testing the effects of using Fido to reduce subclinical depression and anxiety symptoms and compared them with the use of self-help materials from the book “Mind Over Mood” [19].

Based on previous clinical research of chatbots [6,8-10], we aimed to investigate the direct intervention effects and their stability. We hypothesized that, after a 2-week intervention and a 1-month follow-up, the following would occur:

1. The chatbot group reports lower depression, anxiety, and worry symptoms than those with the self-help book only.
2. The chatbot group has higher satisfaction with life than those using the self-help book only.
3. The positive affect is higher, and negative affect is lower than prior to intervention. Furthermore, this change is greater in the chatbot group than in the group with the self-help book.

Based on previous research on the chatbot-user interaction [8,18], we also hypothesized that:

1. Loneliness is lower in the chatbot group than in the group with the self-help book.
2. Participants form a bond with the chatbot, scoring at least 4 on the Working Alliance Inventory-Short Revised (WAI-SR) scale.
3. The users’ assessments of the chatbot's linguistic pragmatics correlate positively with the level of UX.

## Methods

### Trial Design

**Figure 1:**
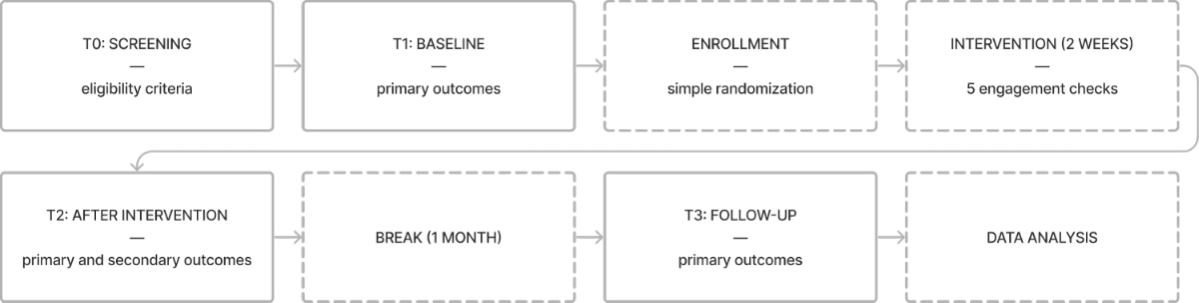
Procedure flowchart with measures and time points (T0-T3).

We used a 2х3 mixed factorial design with 2 intervention arms (Fido chatbot vs self-help book) and 3 time points (before the intervention [T1], immediately after the intervention [T2], and at a follow-up 1 month after the previous measurement [T3]). The primary intervention lasted 2 weeks. After the intervention, the use of the technique was not obligatory (but it was not forbidden). For an overview of the procedure, see Figure 1.

### Participants

Participants were recruited via Facebook and Instagram advertisements from May 2022 to June 2022 and screened using a Qualtrics online survey (measurement at T0). The estimated sample size was 80 participants, with an estimated dropout of around 15%. The sample size was based on previous studies of chatbot interventions [6,9], in which the estimated sample size (ie, N=70) allowed for detecting a moderate-large effect for depression. We increased the sample size to 80 due to the high dropout rate in previous studies (average of 7 participants per group) [6,9].

Eligibility criteria included (1) age between 18 years and 35 years; (2) not undergoing psychotherapy, coaching, nor psychopharmacological treatment; (3) no diagnosis of a neurological disorder; (4) declaring at least mild depressive or anxiety symptoms by achieving a total score of at least 16 points on the Center for Epidemiologic Studies Depression Scale Revised (CESD-R) [20,21] or at least 50 points on the Penn State Worry Questionnaire (PSWQ) [22,23]; and (5) being able to visit the study site in Poznań (Poland) to complete the follow-up measurements. Computer literacy and proficiency in Polish were implicitly presumed as all participants completed an online screening survey written in Polish.

After enrollment, the research team members performed simple randomization with a 1:1 ratio (via Python script). Because the intervention involved either using the chatbot or the book, participants were informed about their assignment. To reduce the effect of expectation bias, we intentionally masked our hypotheses about the superiority of chatbot-supported therapy. The research team members remained unblinded as well.

During the onboarding for the intervention, the participants were asked to use the assigned intervention technique as needed (ie, with no prespecified minimal time of use per day or week). The uptake of the intervention was monitored via regular commitment checks delivered by email, which could serve as a reminder to use the assigned intervention technique.

All participants received compensation for their involvement: Zl 90 (US $22.57) directly after the intervention and Zl 70 (US $17.56) at the follow-up.

### Ethics Approval and Informed Consent

The study received approval from the Ethical Review Board at SWPS University of Social Sciences and Humanities (opinion no. 2022-158) and was registered during participant enrollment in the Open Science Framework (OSF) Registries [24].

We retrospectively registered the protocol and analysis plan under the clinical trial number NCT05762939. Participants were informed about each intervention, step-by-step procedure, their right to withdraw, and research data confidentiality. All participants expressed their consent via a checkbox on a screening survey. Should one of the interventions have proved more beneficial, we ensured the participants from the other arm could gain access to that intervention's materials.

Due to the Messenger chatbot policies [25], the research team members were hypothetically able to access individual participant messages from the chatbot conversations. This limitation is due to the fact that, formally, Messenger chatbots are an interface to send messages to Facebook pages. Participants from the experimental condition were informed of this potential privacy breach via a consent form explicitly presented in the chatbot and Meta’s Data Policy [26].

### Interventions

#### Chatbot Intervention—Fido

For the experimental group, we used a free, prerelease version of a therapy-supporting Polish-language chatbot, called Fido [27], integrated into Facebook Messenger. Participants were added as testers in Meta’s development website and accessed the chatbot via links embedded in individual emails. They were given no special training (apart from the initial email instructions) but were offered technical assistance in case of any problems.

The chatbot uses machine learning models for intelligent user intent detection and close-ended input methods (such as choosing 1 option from a list). It was developed using iterative co-development with focus groups consisting of therapists and potential users (for more information, see [18]). Moreover, external therapists provided quality assurance for all therapeutic methods used by the chatbot, while software engineers used standard testing procedures. During the trial, none of the features underwent any changes.

During the onboarding procedure, participants indicate their gender and receive information on user terms and data protection, as well as basic training in cognitive biases because understanding them is crucial for interaction with Fido. After onboarding, users can try different therapeutic techniques implemented in Fido using a tree-based structure.

The primary functionality of Fido is providing dialogue focused on intelligent recognition of cognitive biases and their subsequent modification using Socratic questioning. It also recognizes suicidal ideation and reacts to it by redirecting users to emergency hotlines.

**Figure 2:**
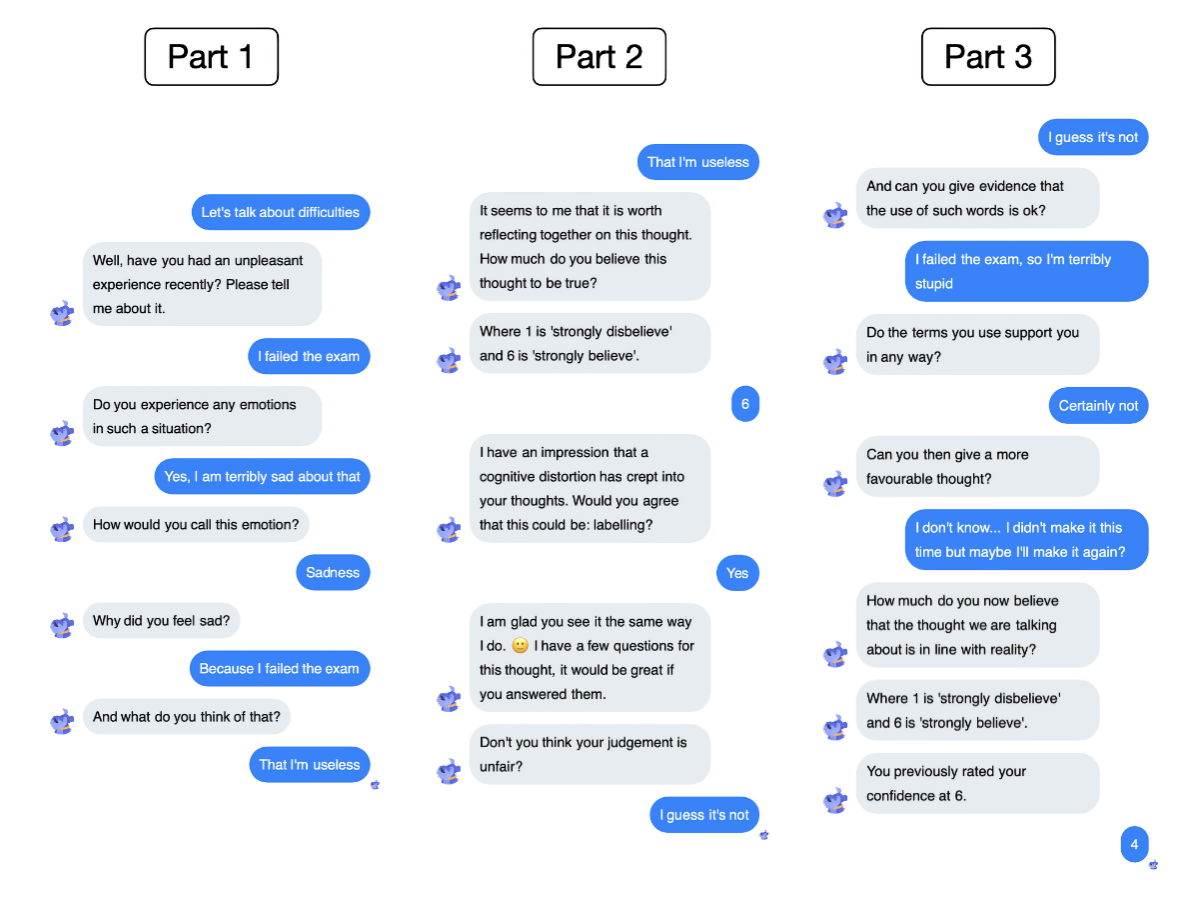
An example of a Fido-patient conversation (translated from Polish to English): ABC technique and cognitive distortion recognition.

To maximize Fido’s performance, one of its machine learning models implements the so-called ABC technique (known from CBT), which helps patients organize and differentiate between activating events, beliefs, and their emotional or behavioral consequences [28], as presented in Figure 2. Fido also provides psychoeducation about depression, anxiety, and emotions. Last, it embraces gratitude practice exercises [19].

#### Control Intervention—Materials From “Mind Over Mood”

Chapters 1-6 and 12 from the Polish translation of “Mind Over Mood” [19,29] were used to familiarize control participants with similar content and tasks as those provided to the experimental group. The book contains psychoeducation content with self-help exercises and is a well-established therapist’s guide to depression.

### Outcomes

#### Primary Outcomes Measures

##### Overview

Primary outcomes were assessed at the 3 time points: T1, T2, T3. All questionnaires were implemented in Qualtrics and administered mostly offline at the study site; when participants could not visit the lab, they were given a link to an online version of the survey. Participants completed validated Polish adaptations of the scales. For primary analyses, we used sum scores of every single primary outcome measured at 1 time point (not change scores).

##### CESD-R

Depression symptom severity was measured using the CESD-R [20,21]. It is a 20-item screening tool for major depressive disorder. The scale uses 9 symptom groups defined in the Diagnostic and Statistical Manual (DSM)-5 [30]: sadness, anhedonia, appetite, sleep, thinking/concentration, guilt, fatigue, agitation, and suicidal ideation.

##### Patient Health Questionnaire-9

Another measure used to assess participants' depression was the Patient Health Questionnaire-9 (PHQ-9) [31,32] from the self-administered version of the Primary Care Evaluation of Mental Disorders (PRIME-MD) inventory [33]. This brief, 9-item scale is based on DSM-IV criteria for depression. It is used mainly for symptom severity monitoring in primary care.

##### PSWQ

We used the PSWQ [22,23] to measure worry tendency, which is the primary component of generalized anxiety disorder. The 16-item scale addresses worry excessiveness, generality, and uncontrollable dimensions.

##### State-Trait Anxiety Inventory

The State-Trait Anxiety Inventory (STAI) [34,35] is another questionnaire used to measure anxiety, both as a temporary state and as a relatively fixed trait of an individual. We used the 20-item trait scale of STAI.

##### Positive and Negative Affect Schedule

The Positive and Negative Affect Schedule (PANAS) [36,37] was used to measure the general affect during the last 2 weeks. We calculated 2 subscale sum scores: 1 for positive feelings (9 items) and 1 for negative affect (9 items). The last 2 items of the original scale were not administered due to human error in the online survey implementation.

##### Satisfaction With Life Scale

Primary measures also included a brief, 5-item Satisfaction With Life Scale (SWLS) [38,39], which was used to assess global life satisfaction in a cognitive-judgmental aspect. Together with positive and negative affect, life satisfaction is an important component of subjective well-being.

##### Revised UCLA Loneliness Scale

We used the 20-item Revised UCLA Loneliness Scale (R-UCLA) [40,41] to assess other specific aspects of well-being, namely subjective feelings of loneliness and social isolation.

#### Secondary and Other Outcome Measures

##### Overview

During the 2-week intervention, participants received 5 engagement check surveys, each containing a single question: “How much time have you spent on therapeutic chatbot/book use during the last 48 hours?” Answers were in 10-minute increments. We calculated the sum score of the engagement checks as the total amount of declared time spent on book or chatbot use in minutes. Each survey had to be completed in less than 24 hours.

After the intervention (T2), all participants also were asked an open-ended question: “How many times have you used the chatbot / read the book in the past two weeks?” If a participant declared a range of values (eg, “10-12 times”), we recoded their answer as the median value in this range.

Another secondary measure used in both arms at T2 was a 12-item test that assessed the participants’ acquired knowledge of psychoeducation topics covered both by Fido and the “Mind Over Mood” book (see Multimedia Appendix 1). The test was administered in a paper-and-pencil format at the study site.

Participants from the chatbot arm were also asked to complete several additional scales at T2, including the WAI-SR and UX.

##### WAI-SR

The WAI-SR [42] is a 12-item scale that measures therapeutic alliance in 3 key areas defined by Bordin [43], which are (1) agreement on the tasks and (2) goals of the therapy and (3) overall patient-therapist affective bond. As agent-guided therapy also requires some form of therapeutic chatbot–user alliance, we adapted this scale to be used in our study. All items were first translated to Polish using a forward and backward translation with reconciliation. Next, we modified phrases related to human-led therapy to increase their relevance for the chatbot therapy (see Multimedia Appendix 2 for the original WAI-SR items and their adaptation for this study). Items from the tasks and goals subscales were not as relevant to this study (because Fido does not establish these elements of the intervention; they are determined ad hoc by the user), so alongside the total average scale score, we also made use of the average of the bond subscale.

##### UX

For the general assessment of UX, we included nonmandatory items from several scales used in other studies: Acceptability E-scale, Human-Agent Interaction Scale (HAIS), Language Pragmaticality Scale (LPS).

The Acceptability E-scale [44] is used to measure the overall acceptability and usability of health-related computer applications. The team prepared its Polish translation using a similar procedure to our adaptation of WAI-SR (see Multimedia Appendix 3 for both the original and the translation). The original scale included 5 items. One of the items regarded the functionality of the program. We used this item 4 times, as we wanted to check the functionality of 4 different techniques (psychoeducation, cognitive bias recognition, suicidal thought recognition, and gratitude practice). This way, we ended up with 9 items and used their sum as a total score.

The HAIS [45] has answers that range from 1 to 7 (see Multimedia Appendix 4). The calculated sums from 6 subscales were used: Supportive Anthropomorphic Traits (3 items), Unsupportive Anthropomorphic Traits (4 items), Behavioral Traits (7 items), Uncanny Valley (6 items), Competence (6 items), Warmth (6 items).

We previously used the LPS [18] to assess the perceived ability of Fido to communicate in a pragmatically sound manner. The scale consists of 4 separately scored items with answers on a percentage scale (0%-100%): “What percentage of overall chatbot statements were adequate?” “What percentage of overall chatbot statements were neutral (neither adequate nor inadequate)?” “What percentage of overall chatbot statements were inadequate?” “In what percentage have you been feeling understood by a chatbot?”

The preregistered plan of the study also included chatbot metadata (frequency of use or time spent in the conversation) for each user after the intervention (T2). However, due to technical and ethical reasons, we could not link the questionnaire data with anonymous chatbot metadata. Therefore, we only used the metadata (frequency of use) at a group level.

### Analytical Methods

For baseline, we conducted contingency analyses on the nominal and ordinal data and 2-tailed Welch *t* tests on the continuous data, to measure whether groups were different in any way at the beginning of the study.

To assess the direct treatment effects on each primary outcome, we conducted repeated measures ANOVA with arm (experimental/control) as a between-group factor, as well as time point (T1/T2) as a within-group factor. We repeated the analyses to test treatment stability, including the scores obtained before versus 1 month after the intervention (T1/T3).

After the intervention (T2), we calculated descriptive statistics on human-agent interaction measures as well as the frequency of use (subjective measures and metadata), declared time spent on the intervention, and knowledge test scores. We also analyzed Pearson correlations between the human-agent interaction measures.

## Results

### Participants

**Figure 3:**
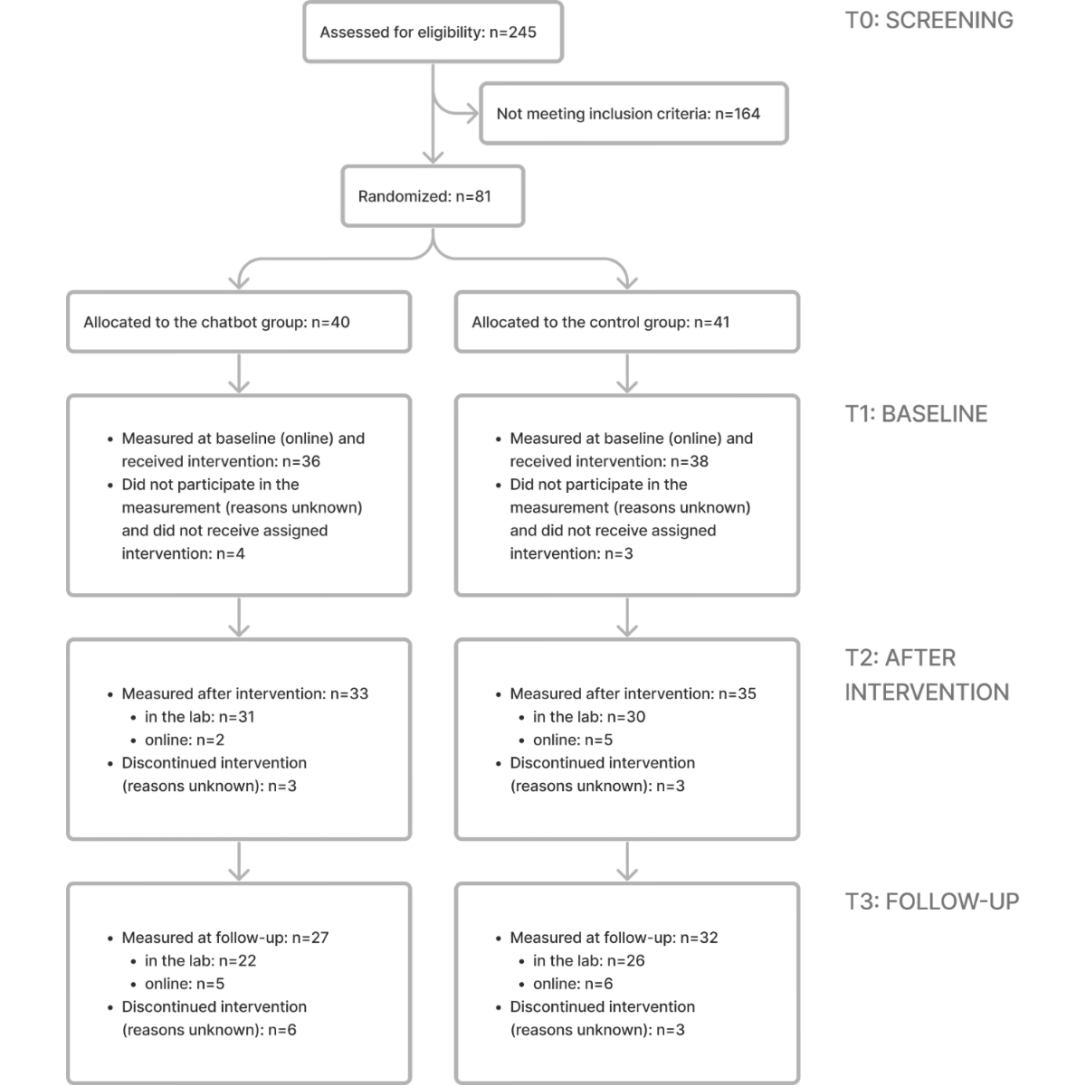
Flow of participants through each stage of the study.

Of the 245 people screened, 81 were admitted to the study. For an overview of participant flow, see the CONSORT (Consolidated Standards of Reporting Trials) diagram (Figure 3). Basic demographic characteristics as well as screening scores (CESD-R and PSWQ at T0) are presented in Table 1. Participants from the chatbot and control conditions did not differ in terms of age, sex, education, employment, university student status, or screening scores (at T0). Moreover, there was no major difference between the groups in any primary outcome measures at baseline (*t*s_72_<1.57, *P*s>.12). Detailed *t* test results are presented in Multimedia Appendix 5.

**Table 1:** Participants’ demographic characteristics and screening scores (Center for Epidemiologic Studies Depression Scale Revised [CESD-R] and Penn State Worry Questionnaire [PSWQ]) as well as *P* values for the between-group tests before randomization (at T0).

Characteristics			Treatment group					*P* value^a^
			Chatbot (n=40)		Control (n=41)			
**Sex, n (%)^b^**								.36
	Female	29 (73)		29 (71)				
	Male	11 (28)		10 (24)				
	Prefer not to say	0		2 (5)				
Age (years), mean (SD)			26.60 (5.06)		24.76 (4.01)		.07	
**Employment status, n (%)^b^**								.75
	Employed	26 (65)		27 (66)				
	Unemployed	7 (2)		5 (12)				
	Other	7 (2)		9 (22)				
**University student status, n (%)^b^**								.65
	Student	19 (48)		23 (56)				
	Not a student	21 (52)		18 (44)				
**Highest level of education, n (%)^b^**								.44
	General secondary school	17 (43)		19 (46)				
	University degree (bachelor’s or higher)	23 (58)		22 (54)				
CESD-R score at T0, mean (SD)			27.58 (13.35)		31 (14.24)		.15	
PSWQ score at T0, mean (SD)			59.78 (10)		59.27 (9.91)		.82	

^a^Calculated using the Pearson χ^2^ test for nominal variables and 2-tailed Welch *t* test for continuous variables.

^b^Percentages were rounded and may exceed 100.

### Efficacy After 2 Weeks

We analyzed the efficacy of the intervention, comparing the data from baseline (T1) and after the 2-week intervention (T2). Only the main effects of time were significant at ɑ=.05. For the between-subject and interaction effect results, see the tables in Multimedia Appendix 5.

For depressive symptoms, there were moderate effects of time. Scores for the CESD-R (*F*_1,66_=62.58, *P*<.001; ω^2^=0.08) and PHQ-9 (*F*_1,66_=34.18, *P*<.001; ω^2^=0.06) decreased in both groups.

In terms of anxiety and worry tendency, there was a small decrease in symptom severity in both groups, as measured with the PSWQ (*F*_1,66_=10.78, *P*=.002; ω^2^=0.01) and STAI (*F*_1,66_=25.87, *P*<.001; ω^2^=0.03).

**Figure 4:**
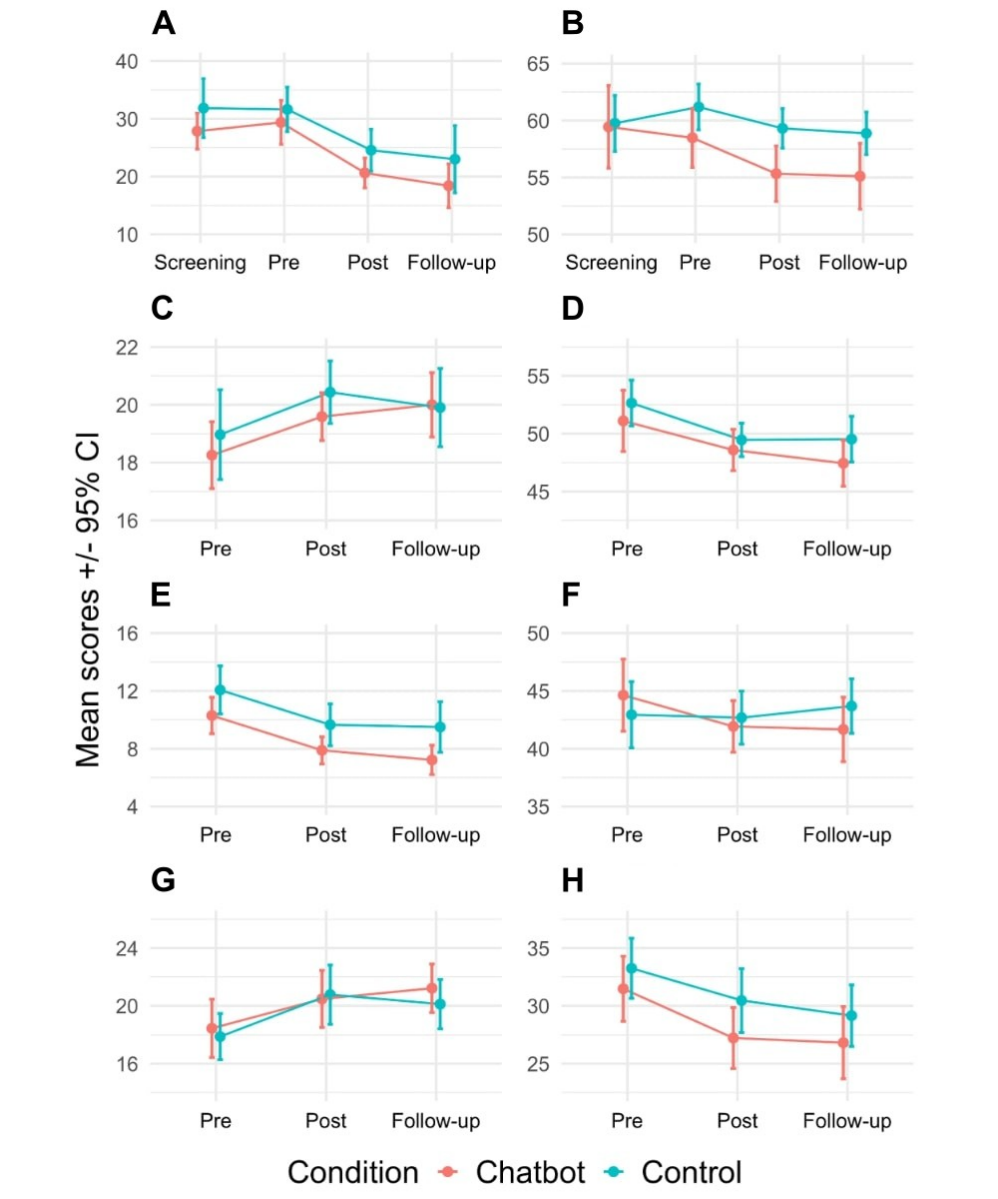
Mean (95% CI) scores at screening, at baseline (Pre), after the intervention (Post), and at the 1-month follow-up for the following primary outcomes: (A) Center for Epidemiologic Studies Depression Scale Revised (CESD-R), (B) Penn State Worry Questionnaire (PSWQ), (C) Satisfaction With Life Scale (SWLS), (D) State-Trait Anxiety Inventory (STAI), (E) Patient Health Questionnaire-9 (PHQ-9), (F) Revised UCLA Loneliness Scale (R-UCLA), (G) Positive and Negative Affect Scale (PANAS)-Positive, (H) PANAS-Negative. Confidence intervals were difference- and correlation-adjusted using the superb R library described by Cousineau et al [46].

For both study arms, we also detected small increases in satisfaction with life (SWLS: *F*_1,66_=13.59, *P*<.001; ω^2^=0.01) and positive affect (PANAS-P: *F*_1,66_=16.54, *P*<.001; ω^2^=0.04), while negative affect decreased (PANAS-N: *F*_1,66_=24.02, *P*<.001; ω^2^=0.05). The decrease in feelings of loneliness was very small and not significant (*F*_1,66_=3.47, *P*=.07; ω^2^=0.00; see Table 2). For a visual representation of treatment efficacy and stability, see Figure 4 [46].

**Table 2:** Primary outcomes at baseline (T1), after the intervention (T2), and at the follow-up (T3), as well as difference scores (T1 vs T2 and T1 vs T3).

Outcome per arm		Score at T1, mean (SD)	Score at T2, mean (SD)	Score at T3, mean (SD)	T1 vs T2, mean difference (95% CI)		T1 vs T3, mean difference (95% CI)	
**CESD-R^a^**						–8.09 (–10.13 to –6.05)		–9.79 (–13.26 to –6.33)
	Chatbot	28.33 (13.84)	20.33 (11.94)	18.41 (13.44)				
	Control	30.74 (13.94)	23.03 (14.30)	23.00 (14.44)				
**PANAS^b^-Positive**						2.56 (1.30 to 3.81)		2.51 (1.21 to 3.82)
	Chatbot	17.89 (6.51)	20.27 (6.29)	21.22 (5.56)				
	Control	17.71 (5.52)	20.66 (7.09)	20.13 (5.88)				
**PANAS-Negative**						–3.83 (–5.39 to –2.27)		–4.38 (–6.26 to –2.50)
	Chatbot	31.19 (7.70)	27.00 (7.26)	26.81 (8.81)				
	Control	32.50 (7.86)	29.77 (8.76)	29.16 (8.46)				
**PHQ-9^c^**						–2.56 (–3.44 to –1.69)		–2.82 (–3.91 to –1.72)
	Chatbot	9.97 (4.98)	7.64 (4.53)	7.22 (4.56)				
	Control	11.87 (5.40)	9.11 (5.59)	9.50 (5.32)				
**PSWQ^d^**						–2.36 (–3.79 to –0.92)		–2.84 (–4.47 to –1.21)
	Chatbot	9.97 (4.98)	7.64 (4.53)	7.22 (4.56)				
	Control	11.87 (5.40)	9.11 (5.59)	9.50 (5.32)				
**R-UCLA^e^**						–1.56 (–3.23 to 0.11)		–1.11 (–3.14 to 0.92)
	Chatbot	45.44 (12.15)	43.06 (13.38)	41.67 (13.08)				
	Control	42.39 (13.25)	41.49 (13.42)	43.69 (12.84)				
**STAI^f^**						–3.31 (–4.61 to –2.01)		–3.40 (–4.99 to –1.80)
	Chatbot	51.86 (8.75)	48.82 (9.25)	47.44 (9.78)				
	Control	52.42 (8.99)	48.54 (8.94)	49.53 (7.96)				
**SWLS^g^**						1.44 (0.66 to 2.22)		1.34 (0.34 to 2.34)
	Chatbot	17.61 (6.67)	18.97 (7.03)	20.00 (7.61)				
	Control	19.50 (5.49)	20.91 (6.07)	19.91 (6.00)				

^a^CESD-R: Center for Epidemiologic Studies Depression Scale Revised.

^b^PANAS: Positive and Negative Affect Scale.

^c^PHQ-9: Patient Health Questionnaire-9.

^d^PSWQ: Penn State Worry Questionnaire.

^e^R-UCLA: Revised UCLA Loneliness Scale.

^f^STAI: State-Trait Anxiety Inventory.

^g^SWLS: Satisfaction With Life Scale.

### Treatment Stability After 1 Month

The measurements from the follow-up (T3) were compared with those at baseline (T1) to determine if previously observed therapeutic effects were stable.

Depressive symptoms remained at a reduced level, as observed in both the CESD-R (*F*_1,57_=32.10, *P*<.001; ω^2^=0.11) and PHQ-9 (*F*_1,57_=26.58, *P*<.001; ω^2^=0.07) scores. A similar effect was found in anxiety (PSWQ: *F*_1,57_=12.14, *P*<.001; ω^2^=0.01) and worry tendency (STAI: *F*_1,57_=18.12, *P*<.001; ω^2^=0.03) scores. Stability in the therapeutic effect was also observed for satisfaction with life (SWLS: *F*_1,57_=7.22, *P*=.009, ω^2^=0.01), positive affect (PANAS-P: *F*_1,57_=14.87, *P*<.001; ω^2^=0.04), and negative affect (PANAS-N: *F*_1,57_=21.67, *P*<.001; ω^2^=0.06). There was not sufficient evidence to conclude that loneliness (as measured using R-UCLA scores) changed from baseline to follow-up (*F*_1,57_=1.19, *P*=.28; ω^2^=0).

As discussed previously, no group and interaction (of group and time) effects were detected for any primary outcomes measures (for detailed results, see Multimedia Appendix 5).

### Human-Agent Interaction Effects

In terms of human-agent interaction characteristics, Fido achieved a mean 2.71 (SD 0.94) points on the WAI-SR scale, with an average score of 3.25 (SD 1.14) on the bond subscale. We detected a moderate correlation of language pragmaticality (item 1: % of messages rated as adequate) with the general UX level measured using the Acceptability E-scale (*r*=0.60, 95% CI 0.32 to 0.78; *P*<.001).

Groups were similar in terms of the frequency of use they reported after the intervention (*t*_63_=1.44, *P=*.15; *d*=0.36, 95% CI –0.13 to 0.84) and scores on the knowledge test (*t*_57_=0.02, *P=*.99; *d*=0.01, 95% CI –0.51 to 0.52). However, the analysis of the time spent on the intervention via regular commitment checks suggested that the control group (mean 117.57, SD 72.40 minutes) actually spent more time reading the book than the chatbot group (mean 79.44, SD 42.96 minutes) spent on the interaction with Fido (*t*_59_=2.75, *P=*.008; *d*=0.64, 95% CI 0.17 to 1.11).

### Exploratory Analyses

We extended our preregistered analyses after gaining insight into the data. The therapeutic alliance score (WAI-SR) correlated positively with the subjective sense of being, as understood from the LPS (*r*=0.79, 95% CI 0.60 to 0.89; *P*<.001), overall acceptability (*r*=0.77, 95% CI 0.57 to 0.88; *P*<.001), and scores for competence (*r*=0.71, 95% CI 0.48 to 0.85; *P*<.001) and behavioral traits (*r*=0.45, 95% CI 0.12 to 0.69; *P=*.01) from the HAIS.

We also detected a moderate, negative correlation between the WAI-SR score and age (*r*=–0.37, 95% CI –0.63 to –0.03; *P*=.04), which motivated us to split the experimental group into 2 subgroups by median (younger and older participants) and compare them using the Student *t* test. Younger participants formed a stronger human-chatbot bond (mean 3.59, SD 0.97) than older participants (mean 2.79, SD 1.23; *t*_31_=2.11, *P=*.04; *d*=0.74, 95% CI 0.02 to 1.45).

**Figure 5:**
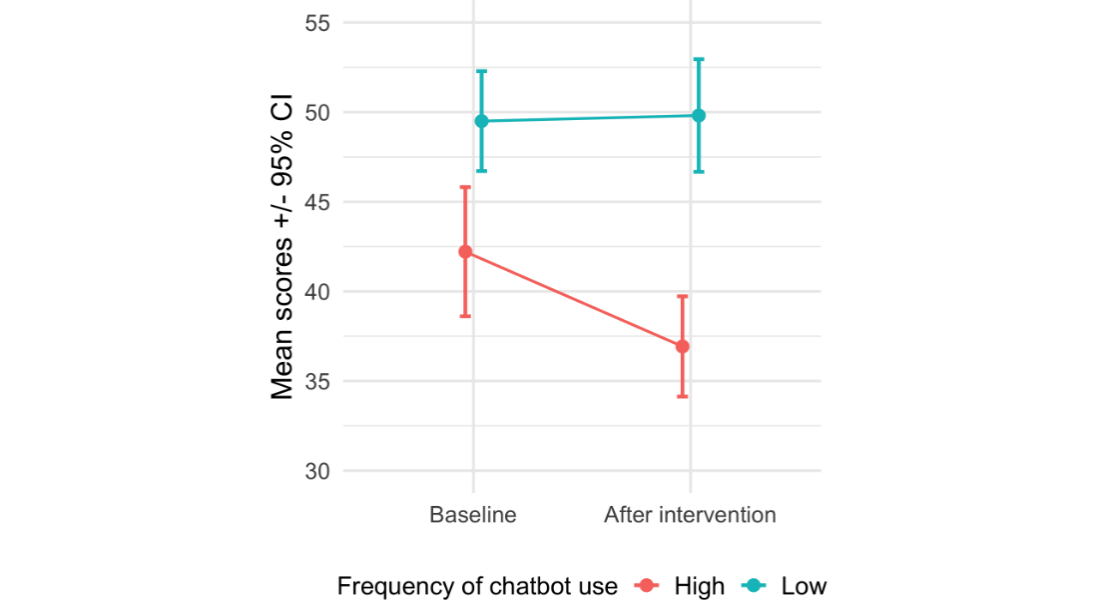
Revised UCLA Loneliness Scale (R-UCLA) scores for the high-frequency and low-frequency users at baseline and after the intervention. Confidence intervals were difference- and correlation-adjusted using the superb R library described by Cousineau et al [46].

Moreover, in the chatbot group, loneliness (R-UCLA) scores after the intervention (at T2) were negatively correlated with the declared frequency of use (*r*=–0.48, 95% CI –0.71 to –0.16; *P*=.006). This finding motivated us to split the observations by median into subgroups of high- and low-frequency chatbot users. Repeated measures ANOVA of the R-UCLA scores with low-frequency and high-frequency users as the between-subjects effect and 2 time points (T1-T2) as the within-subjects effect yielded a statistically significant interaction of frequency and time point (*F*_1,66_=7.417, *P*=.01; ω^2^=0.01; see Figure 5). After 2 weeks, only high-frequency users reported lower levels of loneliness (*t*_16_=3.69, *P*_Bonf._=.005; *d*=0.44, 95% CI 0.07 to 0.81).

### Qualitative Feedback

The team also collected informal, qualitative data through individual in-depth interviews and an open-ended survey question after the intervention (T2). Volunteers (n=16) from the chatbot group answered questions about the perceived advantages and pitfalls of Fido, as well as other unintended effects that had not been assessed by the previously administered questionnaires. The most common response about the negative aspects of using Fido was that there were many failures in recognizing the user’s intent, which resulted in the chatbot telling the user that it did not know what they are talking about. Regarding the positive side effects, the most prominent was that using Fido encouraged the user to make a decision about starting traditional therapy.

## Discussion

### Principal Findings

This study is one of the few that presents an RCT of chatbot therapy efficacy in comparison with an active control, to the best knowledge of authors—the first one for the Polish language. Fido was effective in terms of reducing depression, anxiety, and worry symptoms, as well as for increasing satisfaction with life during the 2-week intervention. We also detected an increase in positive affect, along with a decrease in negative affect, after the intervention using Fido. All effects were stable for at least one month following the interventions. In contrast to some previous findings [6] about agent-guided therapy, we did not detect expected differences in the effectiveness between the intervention based on Fido and the one in which the book chapters were used.

Although there were no between-group effects in terms of symptoms or affect, or for obtained psychoeducational knowledge or the frequency of use, we did detect some other differences. Worth consideration is that the chatbot users spent less time on the intervention than the control group. Thus, although the effects of working with Fido were comparable to those achieved using self-help materials, they were achieved in a shorter period of time, which is an obvious benefit considering the concentration and motivation problems of patients in mental health crises. The differences in time spent on the 2 interventions may be related to differences in the form of the presented content: Even though both techniques are text-based, reading an ebook may be challenging, in contrast to absorbing shorter messages from the chatbot.

Contrary to our expectations, we did not detect a superior decrease in loneliness levels in the chatbot group when compared with the control group. However, we observed a drop in loneliness among participants who used the chatbot with higher frequency, an effect that did not occur either among participants who used Fido with low frequency or in the control group. Interestingly, although loneliness is linked to well-being, we did not detect a decrease in loneliness in the control group, but we did observe a therapeutic effect on all other primary outcome variables. In the face of this result, it is difficult to relate a decline in loneliness observed in high-frequency chatbot users to the decrease in depression or anxiety. A dissociation between treatment effects on loneliness *versus* anxiety and depression was observed in a study investigating the efficacy of internet-delivered CBT [47]. Some authors consider loneliness as a transdiagnostic phenomenon that can vary in intensity across different diagnoses [48]. It is possible that loneliness was differentially represented as a factor related to depressiveness and anxiety in our study sample, and we, therefore, did not observe a stable relationship between the effects observed for loneliness and those for the anxiety and depression levels. It is also possible that the duration of our study was simply too short to capture this relationship, as changes in levels of loneliness and depression seem to occur with different temporal dynamics [49]. Future studies should try to track the effects of therapy chatbots for longer time periods and replicate our findings regarding loneliness using nontherapeutic agents, as the interaction with such chatbots may also have a direct effect on loneliness with or without any effect on the mental health condition.

Younger participants formed a stronger relationship with Fido, bonding at an average score of 3.59 (on a 1-to-5 positively scored scale), which is higher than the bond reported for internet interventions but lower than Woebot or human-involved therapy (Woebot formed a bond at 3.8, traditional CBT formed a bond at 4, and group CBT formed a bond at 3.8 on the same scale [8]). Participants used the chatbot as frequently as the book in the 2-week intervention period, but the book readers declared spending more time using their psychoeducation materials than chatbot users.

### Comparison With Prior Work and Limitations

We did not replicate the findings from the study on Woebot that demonstrated better treatment effects for the chatbot-based intervention than those for the self-help materials [6]. In addition, the alliance built with Woebot was higher than with Fido. This may suggest that, at this stage, Fido is less effective and user-friendly than Woebot.

However, we would argue that the lack of group differences in this study do not result from Fido's insufficiency but is rather related to the fairly high effectiveness of the self-help materials that we used and the differences in our experimental procedure. First, the materials used in the study by Fitzpatrick et al [6] were mostly psychoeducation, and the materials used in the previous study required not only getting acquainted with psychoeducation but also performing a few cognitive exercises. Second, the materials used for the control group in the Woebot study were provided by the WHO, and those included in our study were derived from a CBT handbook that was empirically proven to be efficient [50]. Third, our study differed in the experimental protocol from the Woebot protocol; that is, in our study, engagement checks were sent to participants every 3 days, which may have served as a reminder to use the intervention. Although it provided valuable insight into participants’ behaviors, it may have also increased the treatment effects, making the 2 studies difficult to compare.

Even though we did not replicate the outcomes from the Woebot study [6], our data are in line with the results of other studies on digital therapeutics. Studies of mental health–supporting applications yield high efficacy mostly when compared with waiting list control samples but not with active control groups [3]. The lack of differences between the 2 groups investigated in this study prompts us to ask whether the interventions that we used would also be more efficacious when compared with a passive control group (ie, waiting control) and if they would match traditional CBT. To gain a deeper understanding of the patient-reported outcomes, further assessment methods could be used, such as clinical interviews [3] or even psychophysiological methods, that were previously used for human-agent interaction studies [45].

Another important factor when we consider the lack of replication of previous effects is the kind of intervention used in our control group, as different effect sizes are reported depending on the kind of intervention. In our study, the well-established handbook materials were used for the control condition, and the procedure ensured that participants used them regularly, which might have increased the effects of the control intervention [3]. It seems important for the entire area of intervention research to systematize the issue of the control condition in the future and to enable thoroughly selected active control conditions to a greater extent.

There are some other limitations. Our experimental procedure does not allow us to completely exclude the influence of the time factor: It may be that all participants improved only as a result of the passing time or regression toward the mean. The use of a waiting control group could enable control over this aspect of our study, providing information about differences in the outcome data without any intervention. However, if we compare the recruitment and baseline data, we can see that waiting for the beginning of treatment did not change the levels of depression and worry, which suggests that the overall affect and well-being were stable over time and changed only after the intervention.

Last, some elements of this study would be different in a routine application setting. The standard use of Fido or other self-help materials may not include frequent commitment checks or psychological assessments and certainly would not involve financial compensation. All of these factors could have influenced intervention uptake, effectiveness, and side effects. The generalizability of the results is also limited by the fact that subsequent free versions of Fido will use improved machine learning models (ie, achieving better precision and recall scores in intent recognition) while offering the same set of conversation functionalities. However, the level of human involvement and support should be very similar in the standard use of Fido.

### Future Research

Prospective studies should try to replicate the effects presented here. One future direction would be to extend the timing of the intervention, as 2 weeks are relatively short. Thus, it would be beneficial to conduct at least a 4-week intervention and a 3- or 6-month follow-up to assess whether the effects are sustained after a longer period of time. Furthermore, future studies should consider using other control groups such as waiting lists or traditional CBT.

This article focused mainly on the therapeutic effects; however, during this experiment, we used several measures linked to human-chatbot interaction, which may be further explored. Future studies may provide insight not only into the quantitative measures linked to the use of chatbots but also into qualitative measures, as well as deep analysis of metadata, such as the distribution of cognitive biases and their relationship with the mental health condition. Those studies may extend our knowledge linked to digital therapeutics and provide a theoretical background for the development of further therapy applications.

### Data Availability

The data sets generated during and/or analyzed during this study are available in the Open Science Framework repository [24].

## Appendix

Multimedia Appendix 1 Psychoeducation Knowledge Test.

Multimedia Appendix 2 Working Alliance Inventory-Short Revised.

Multimedia Appendix 3 Acceptability E-scale.

Multimedia Appendix 4 Human-Agent Interaction Scale.

Multimedia Appendix 5 Detailed Results.

Multimedia Appendix 6 CONSORT-EHEALTH checklist (V 1.6.1).

## Abbreviations

AG-CBT: agent-guided cognitive behavioral therapy
CESD-R: Center for Epidemiologic Studies Depression Scale Revised
CONSORT: Consolidated Standards of Reporting Trials
DSM: Diagnostic and Statistical Manual
HAIS: Human-Agent Interaction Scale
LPS: Language Pragmaticality Scale
OSF: Open Science Framework
PANAS: Positive and Negative Affect Scale
PHQ-9: Patient Health Questionnaire-9
PRIME-MD: Primary Care Evaluation of Mental Disorders
PSWQ: Penn State Worry Questionnaire
RCT: randomized controlled trial
R-UCLA: Revised UCLA Loneliness Scale
STAI: State-Trait Anxiety Inventory
SWLS: Satisfaction With Life Scale
T0: recruitment
T1: baseline
T2: immediately after the 2-week intervention
T3: 1-month follow-up
UX: user experience
WAI-SR: Working Alliance Inventory-Short Revised
WHO: World Health Organization
