# Supplementary material for: Effectiveness of a Web-based and Mobile Therapy Chatbot on Anxiety and Depressive Symptoms in Subclinical Young Adults: Randomized Controlled Trial
Source: JMIR Form Res. 2024 Mar 20;8:e47960. doi: 10.2196/47960 (PMC10993129; doi:10.2196/47960)
Supplement: Multimedia Appendix 1 [file formative_v8i1e47960_app1.pdf]

APPENDIX 1

Psychoeducation Knowledge Test

	ORIGINAL (PL)	TRANSLATION (ENG)
	Poniżej znajduje się kilka pytań, na które poprosimy, aby Pan(i) odpowiedział(a). Poprosimy przy każdym zaznaczyć odpowiedź, którą uważa Pan(i) za poprawną. Jeśli nie wie Pan(i), która jest poprawna, proszę nic nie zaznaczać. Test nie sprawdza Pana/Pani umiejętności, tylko to, jak dana forma interwencji jest skuteczna w nauczaniu treści psychoedukacyjnych.	Below are some questions that we ask you to answer. For each one, please mark the answer you think is correct. If you don't know which one is correct, please don't mark anything. The test does not test your skills, but only the effectiveness of your intervention in teaching psychoeducational content.
1	Pani Agata nakrzyczała na swojego chłopaka, gdy ten spóźnił się z pracy do domu. Zgodnie z modelem zależności między emocjami, myślami i zachowaniem najbardziej bezpośrednią przyczyną jej zachowania była:<br>1. Myśl<br>2. Emocja<br>3. Sytuacja, w której się znalazła<br>4. Odpowiedzi A i B są prawidłowe	Ms. Agatha yelled at her boyfriend when he was late home from work . According to the model of the relationship between emotions, thoughts and behavior, the most direct cause of her behavior was:<br>1. Thought<br>2. Emotion<br>3. The situation in which she found herself<br>4. Answers A and B are correct
2	Pan Marek otrzymał złą ocenę okresową i pomyślał sobie *Jestem beznadziejny. Nic mi nie wychodzi*. Jeżeli wierzy w swoje myśli, to można spodziewać się, że w jej wyniku doświadczył następującej emocji:<br>1. Radości<br>2. Smutku<br>3. Złości<br>4. Zaskoczenia	Mr. Mark received a bad interim evaluation and thought to himself *I'm hopeless. Nothing works for me.* If he believes his thoughts, it can be expected that he experienced the following emotion as a result:<br>1. Joy<br>2. Sadness<br>3. Anger<br>4. Surprise
3	Pani Jola i Pani Małgosia wygrały 1000 zł w lotto. Pani Ola się ucieszyła, a Pani Małgosia wściekła. Która z poniższych myśli mogła pojawić się u Pani Małgosi?<br>1. To niesprawiedliwe, że wygrałam tylko 1000 zł<br>2. Będę mogła wreszcie wyjechać na wakacje<br>3. Nareszcie się udało<br>4. Szkoda, że nie wygrałam 1500 zł	Mrs. Jolene and Mrs. Margaret won PLN 1,000 in the lottery. Mrs. Jolene was happy and Mrs. Margaret was furious. Which of the following thoughts might have occurred to Mrs. Margaret?<br>1. It's unfair that I won only PLN 1,000<br>2. I will finally be able to go on vacation<br>3. I finally made it<br>4. It's a pity that I didn't win PLN 1,500
4	Terapię poznawczo-behawioralną możemy stosować, gdy:<br>1. Mamy problemy z samopoczuciem, relacjami i nastrojem<br>2. Chcemy zadbać o rozwój swojej osobowości<br>3. Mamy zaburzenia odżywiania, problem z alkoholem i narkotykami<br>4. Wszystkie powyższe odpowiedzi są poprawne	We can use cognitive-behavioral therapy when:<br>1. We have mood, relationship and mood problems<br>2. We want to take care of our personality development<br>3. We have eating disorders, problems with alcohol and drugs<br>4. All of the above answers are correct

ORIGINAL (PL)		TRANSLATION (ENG)
5	Terapia poznawczo behawioralna, podchodząc do myśli, wskazuje że:<br>1. Trzeba myśleć pozytywnie<br>2. Myśli pomagają nam zrozumieć emocje, jakich doświadczamy<br>3. Kiedy przestajemy myśleć, czujemy się lepiej<br>4. Myśli są ważne, bo pokazują nam rzeczywistość taką jaką jest	When it comes to thoughts, the cognitive-behavioral therapy indicates that:<br>1. It is necessary to think positively<br>2. Thoughts help us understand the emotions we experience<br>3. When we stop thinking, we feel better<br>4. Thoughts are important because they show us reality as it is
6	Do kryteriów depresji można zaliczyć:<br>1. Obniżony nastrój lub brak ochoty na aktywności, które dotychczas sprawiały przyjemność<br>2. Brak snu lub nadmierną senność<br>3. Przynajmniej dwutygodniowy okres trwania symptomów<br>4. Wszystkie powyższe odpowiedzi są prawdziwe	Criteria for depression can include:<br>1. Lowered mood or lack of desire for activities that previously gave pleasure<br>2. Lack of sleep or excessive sleepiness<br>3. At least a two-week duration of symptoms<br>4. All of the above answers are true
7	Odczuwanie lęku:<br>1. Zawsze świadczy o zaburzeniu wymagającym interwencji<br>2. Jest naturalnym elementem życia<br>3. Jest niemożliwe do zauważenia<br>4. Zawsze świadczy o nadchodzącym niebezpieczeństwie	Perception of anxiety:<br>1. Is always indicative of a disorder that requires an intervention<br>2. Is a natural part of life<br>3. Is impossible to notice<br>4. Is always indicative of imminent danger
8	Praktykowanie wdzięczności ma na celu przede wszystkim:<br>1. Nauczyć nas, że każda trudność nas wzmacnia<br>2. Nauczyć nas doceniania miłych rzeczy, które nas spotykają<br>3. Nauczyć nas doświadczania tylko dobrych emocji<br>4. Nauczyć nas przekraczania własnych słabości	Practicing gratitude is primarily intended to:<br>1. Teach us that every difficulty strengthens us<br>2. Teach us to appreciate the nice things that happen to us<br>3. Teach us to experience only good emotions<br>4. Teach us to transcend our own weaknesses
9	Magda przeglądała Instagram i znalazła zdjęcie dwóch starych przyjaciółek. Pomyślała *Spotkały się beze mnie, bo pewnie nie chcą mnie znać* i poczuła smutek. Co Magda powinna w tej sytuacji zrobić, zgodnie z duchem terapeutycznym?<br>1. Zastanowić się, jakie są dobre strony tej sytuacji i być za nie wdzięczna<br>2. Zastanowić się, czy ta myśl jest prawdziwa i ją spróbować ją podważyć<br>3. Zastanowić się, czy te dwie osoby nadal zasługują na miano jej przyjaciółki<br>4. Zauważyć swoje emocje i dać im upust	Magdalene was browsing through Instagram and found a photo of two old friends. She thought *They met without me, because they probably don't want to know me* and felt sad. What should Magdalene do in this situation, according to the therapeutic spirit?<br>1. Reflect on the good things about this situation and be grateful for them<br>2. Reflect on whether this thought is true and try to challenge it<br>3. To consider whether these two people still deserve to be called her friend<br>4. To notice her emotions and give vent to them

	ORIGINAL (PL)	TRANSLATION (ENG)
10	*Konrad spodziewa się, że koledzy znienawidzili go za jego zachowanie.* To zdanie wskazuje na to, że z największym prawdopodobieństwem u Konrada pojawiła się<br>1. Emocja lęku<br>2. Emocja złości<br>3. Unikanie<br>4. Niekoniecznie słuszna myśl zwana *czytaniem w myślach*	*Konrad expects that his colleagues hated him for his behavior*. This sentence indicates that, in all likelihood, Konrad has developed:<br>1. An emotion of fear<br>2. An emotion of anger<br>3. An avoidance<br>4. A not-necessarily-right belief called *mind reading*
11	Które z poniższych pytań jest najlepiej nacelowane na podważenie przekonania *To nie może się udać*.<br>1. Skąd wiesz, że tak pomyśleli?<br>2. Jesteś pewien, że możesz przewidzieć przyszłość?<br>3. Czy nie stawiasz sobie czasem zbyt wysokich wymagań?<br>4. Skąd pewność, że Twoje uczucia mówią prawdę?	Which of the following questions is best aimed at challenging the belief *It can't work*:<br>1. How do you know they thought that?<br>2. Are you sure you can predict the future?<br>3. Do you sometimes set your expectations too high?<br>4. How can you be sure that your feelings are telling the truth?
12	Po zakończeniu stawiania pytań sprawdzających słuszność myśli warto ponownie sprawdzić przede wszystkim:<br>1. Sytuację wyjściową<br>2. Nastrój lub wiarę w sprawdzaną myśl<br>3. Reakcję partnera interakcji<br>4. Nasilenie symptomów depresji	After you have finished posing questions to check the validity of your thoughts, it is worth re-checking first of all:<br>1. The initial situation<br>2. The mood or belief in the thought being checked<br>3. The reaction of the interaction partner<br>4. Severity of depressive symptoms
