# Supplementary material for: Effectiveness of a Web-based and Mobile Therapy Chatbot on Anxiety and Depressive Symptoms in Subclinical Young Adults: Randomized Controlled Trial
Source: JMIR Form Res. 2024 Mar 20;8:e47960. doi: 10.2196/47960 (PMC10993129; doi:10.2196/47960)
Supplement: Multimedia Appendix 2 [file formative_v8i1e47960_app2.pdf]

APPENDIX 2

Working Alliance Inventory-Short Revised (WAI-SR)[3]

ORIGINAL (ENG)		ADAPTATION USED IN THE STUDY (PL)
Below is a list of statements and questions about experiences people might have with their therapy or therapist. Some items refer directly to your therapist with an underlined space – as you read the sentences, mentally insert the name of your therapist in place of ___ in the text. Think about your experience in therapy, and decide which category best describes your own experience.		Poniżej znajduje się lista stwierdzeń dotyczących doświadczeń, jakie ludzie mogą mieć z terapią prowadzoną przez chatboty. Pomyśl o swoich doświadczeniach z terapią prowadzoną przez chatbota Fido i zdecyduj, która kategoria najlepiej opisuje Twoje doświadczenia.
1	As a result of these sessions I am clearer as to how I might be able to change.	Dzięki rozmowom z chatbotem czuję, że mam większą jasność co do tego, jak mogę się zmienić.
2	What I am doing in therapy gives me new ways of looking at my problem.	Rozmowy z chatbotem dają mi nowe sposoby patrzenia na mój problem.
3	I believe___likes me.	Myślę, że chatbot Fido mnie lubi.
4	___and I collaborate on setting goals for my therapy.	Fido i ja współpracujemy w ustalaniu celów mojej terapii.
5	___and I respect each other.	Fido i ja szanujemy się nawzajem.
6	___and I are working towards mutually agreed upon goals.	Fido i ja dążymy do wspólnie ustalonych celów.
7	I feel that___appreciates me.	Czuję, że Fido mnie docenia.
8	_____ and I agree on what is important for me to work on.	Fido i ja zgadzamy się w sprawie tego, nad czym powinnam/powinienem pracować.
9	I feel _____ cares about me even when I do things that he/she does not approve of.	Myślę, że Fido troszczy się o mnie nawet wtedy, gdy robię rzeczy, których on nie pochwala.
10	I feel that the things I do in therapy will help me to accomplish the changes that I want.	Czuję, że to, co robię na terapii z Fido, pomoże mi w dokonaniu zmian, których pragnę.
11	_____ and I have established a good understanding of the kind of changes that would be good for me.	Fido i ja dobrze doszliśmy do tego, jakiego rodzaju zmiany byłyby dla mnie korzystne.
12	I believe the way we are working with my problem is correct.	Uważam, że sposób, w jaki pracujemy nad moim problemem, jest prawidłowy.

RESPONSE OPTIONS:

1 – rzadko (rarely), 2 – czasami (sometimes), 3 – dość często (quite often), 4 – bardzo często (very often), 5 – zawsze (always)

SUBSCALES:

Goals (items 4, 6, 8, 11), Tasks (items 1, 2, 10, 12), Bond (items 3, 5, 7, 9)

[3] Munder T, Wilmers F, Leonhart R, Linster HW, Barth J. Working Alliance Inventory-Short Revised (WAI-SR): psychometric properties in outpatients and inpatients. Clin Psychol Psychother 2009;231–239. doi: 10.1002/cpp.658
