# Supplementary material for: Effectiveness of a Web-based and Mobile Therapy Chatbot on Anxiety and Depressive Symptoms in Subclinical Young Adults: Randomized Controlled Trial
Source: JMIR Form Res. 2024 Mar 20;8:e47960. doi: 10.2196/47960 (PMC10993129; doi:10.2196/47960)
Supplement: Multimedia Appendix 3 [file formative_v8i1e47960_app3.pdf]

APPENDIX 3

Acceptability E-scale[2]

ORIGINAL (ENG)		ADAPTATION USED IN THE STUDY (PL)
1	How easy was this computer program for you to use? (1: very difficult, 5: very easy)	Jak łatwy był dla Ciebie w użyciu ten chatbot?
2	How understandable were the questions?	Jak zrozumiałe były pytania, które zadawał chatbot?
3	How much did you enjoy using this computer program?	Jak bardzo przyjemne było dla Ciebie korzystanie z chatbota?
4	How helpful was this computer program in describing your symptoms and quality of life?	Jak bardzo skuteczny był chatbot w wykrywaniu zniekształceń poznawczych?
5		Jak bardzo skuteczny był chatbot w wykrywaniu myśli samobójczych?
6		Jak bardzo pomocny był chatbot w dostarczaniu wiedzy dotyczącej ludzkiej psychiki?
7		Jak bardzo pomocny był chatbot w dostarczaniu praktyki wdzięczności?
8	Was the amount of time it took to complete this computer program acceptable?	Czy czas, jaki zajęła interakcja z chatbotem, jest akceptowalny?
9	How would you rate your overall satisfaction with this computer program?	Jak oceniasz ogólną satysfakcję z tego chatbota?

RESPONSE OPTIONS:

1 to 5 (ascending)

[2] Tariman JD, Berry DL, Halpenny B, Wolpin S, Schepp K. Validation and testing of the Acceptability E-scale for Web-based patient-reported outcomes in cancer care. Applied Nursing Research 2011 Feb;24(1):53–58. doi: 10.1016/j.apnr.2009.04.003
