# Supplementary material for: Effectiveness of a Web-based and Mobile Therapy Chatbot on Anxiety and Depressive Symptoms in Subclinical Young Adults: Randomized Controlled Trial
Source: JMIR Form Res. 2024 Mar 20;8:e47960. doi: 10.2196/47960 (PMC10993129; doi:10.2196/47960)
Supplement: Multimedia Appendix 4 [file formative_v8i1e47960_app4.pdf]

APPENDIX 4

Human-Computer Interaction Scale[1]

Proszę ocenić, w jakim stopniu zgadza się Pan(i) ze stwierdzeniem, że chatbot jest:
Please rate to what extent you agree with the statement that the chatbot is:

1. Miły	Kind
2. Taktowny	Considerate
3. Sympatyczny	Likeable
4. Przebiegły	Deceitful
5. Zakłopotany	Ashamed
6. Zazdrosny	Jealous
7. Twórczy	Creative
8. Agresywny	Aggressive
9. Zwinny	Agile
10. Aktywny	Active
11. Energiczny	Energetic
12. Strachliwy	Timid
13. Ospały	Sluggish
14. Silny fizycznie	Physically strong
15. Atrakcyjny	Attractive
16. Dziwny	Odd
17. Familiarny	Familiar
18. Ludzki	Human
19. Przyjazny	Friendly
20. Znany	Well known
21. Kompetentny	Competent
22. Zdolny	Capable
23. Inteligentny	Intelligent
24. Sprawny	Efficient
25. Zręczny	Skilled
26. Pewny siebie	Confident
27. Ciepły	Warm
28. Łagodny	Gentle
29. Szczery	Honest
30. Przyjazny	Friendly
31. O dobrych intencjach	Well intended
32. Godny zaufania	Trustworthy

RESPONSE OPTIONS:
1 (zdecydowanie się nie zgadzam strongly disagree) to 6 (zdecydowanie się zgadzam strongly agree)

SUBSCALES:
Supportive Anthropomorphic Traits (items 1-3), Nonsupportive Anthropomorphic Traits (items 4-7), Behavioral Traits (items 9-14), Uncanny Valley (items 15–20), Competence (items 21–26), Warmth (items 27–32)

[1] Source: Ciechanowski L, Przegalińska A, Magnuski M, Gloor P. In the shades of the uncanny valley: An experimental study of human–chatbot interaction. Future Generation Computer Systems 2019 Mar;92:539–548. doi: 10.1016/j.future.2018.01.055
