# Supplementary material for: Effectiveness of a Web-based and Mobile Therapy Chatbot on Anxiety and Depressive Symptoms in Subclinical Young Adults: Randomized Controlled Trial
Source: JMIR Form Res. 2024 Mar 20;8:e47960. doi: 10.2196/47960 (PMC10993129; doi:10.2196/47960)
Supplement: Multimedia Appendix 5 [file formative_v8i1e47960_app5.pdf]

Detailed Results

All analyses were performed using JASP (Version 0.16.3).

1 Independent samples Welch *t*-test results for primary outcomes measures at T1 (baseline)

Scale	t	df	P value
CESD-R	−0.74	71.84	0.46
PHQ-9	−1.57	71.95	0.12
PSWQ	−0.71	71.89	0.48
STAI	−0.27	71.95	0.79
PANAS Positive	0.13	68.77	0.90
PANAS Negative	−0.72	71.92	0.47
SWLS	−1.33	67.90	0.19
R-UCLA	1.03	71.93	0.31

2 Cronbach's alpha coefficients for scales used at T2 (directly after the intervention)

Scale	No. of items	Cronbach's α	Mean	SD
CESD-R	20	0.92	21.36	13.24
PHQ-9	9	0.85	8.30	5.15
PSWQ	16	0.92	53.67	13.28
STAI	20	0.88	49.36	11.07
PANAS Positive	7	0.92	20.09	7.05
PANAS Negative	11	0.87	27.93	8.73
SWLS	5	0.87	19.67	6.90
R-UCLA	20	0.94	56.79	14.87
WAI-SR	12	0.94	16.16	18.66
Acceptability E-scale	90	0.77	14.80	15.80
HCIS: Supportive Anthropomorphic Traits	3	0.77	7.99	8.41
HCIS: Unsupportive Anthropomorphic Traits	4	0.52	4.49	5.33
HCIS: Behavioral Traits	7	0.78	8.76	10.14
HCIS: Uncanny Valley	6	0.60	10.60	11.55
HCIS: Competence	6	0.91	11.56	13.08
HCIS: Warmth	6	0.87	15.06	16.07

3 Intervention efficacy: RM ANOVA with T1 (baseline) vs T2 (directly after the intervention)

3.1 CESD-R

Within Subjects Effects

Predictor	Sum of Squares	*df*	Mean Square	*F*	*P* value	ω^2
Time	2222.38	1	2222.38	62.583	$< .001$	0.082
Time × Arm	34.15	1	34.15	0.962	0.330	0.000
Residuals	2343.74	66	35.51			

Between Subjects Effects

Predictor	Sum of Squares	*df*	Mean Square	*F*	*P* value	ω^2
Arm	97.33	1	97.33	0.295	0.589	0.000
Residuals	21768.17	66	329.82			

3.2 PHQ-9

Within Subjects Effects

Predictor	Sum of Squares	*df*	Mean Square	*F*	*P* value	ω^2
Time	222.810	1	222.810	34.184	$< .001$	0.057
Time × Arm	0.193	1	0.193	0.030	0.864	0.000
Residuals	430.190	66	6.518			

Between Subjects Effects

Predictor	Sum of Squares	*df*	Mean Square	*F*	*P* value	ω^2
Arm	66.83	1	66.83	1.424	0.237	0.003
Residuals	3097.94	66	46.94			

3.3 PSWQ

Within Subjects Effects

Predictor	Sum of Squares	*df*	Mean Square	*F*	*P* value	ω^2
Time	188.47	1	188.47	10.779	0.002	0.011
Time × Arm	11.59	1	11.59	0.663	0.419	0.000
Residuals	1154.03	66	17.49			

Between Subjects Effects

Predictor	Sum of Squares	*df*	Mean Square	*F*	*P* value	ω^2
Arm	211.7	1	211.7	0.959	0.331	0.000
Residuals	14566.5	66	220.7			

3.4 STAI

Within Subjects Effects

Predictor	Sum of Squares	*df*	Mean Square	*F*	*P* value	ω^2
Time	372.078	1	372.078	25.870	$< .001$	0.032
Time × Arm	0.019	1	0.019	0.001	0.971	0.000
Residuals	949.238	66	14.382			

Between Subjects Effects

Predictor	Sum of Squares	*df*	Mean Square	*F*	*P* value	ω^2
Arm	3.040	1	3.040	0.021	0.886	0.000
Residuals	9659.571	66	146.357			

3.5 SWLS

Within Subjects Effects

Predictor	Sum of Squares	*df*	Mean Square	*F*	*P* value	ω^2
Time	70.509	1	70.509	13.592	$< .001$	0.012
Time × Arm	0.009	1	0.009	0.002	0.967	0.000
Residuals	342.373	66	5.187			

Between Subjects Effects

Predictor	Sum of Squares	*df*	Mean Square	*F*	*P* value	ω^2
Arm	126.3	1	126.29	1.698	0.197	0.005
Residuals	4908.2	66	74.37			

3.6 PANAS Positive

Within Subjects Effects

Predictor	Sum of Squares	*df*	Mean Square	F	P value	ω^2
Time	222.058	1	222.058	16.538	$< .001$	0.037
Time × Arm	0.175	1	0.175	0.013	0.909	0.000
Residuals	886.207	66	13.427			

Between Subjects Effects

Predictor	Sum of Squares	*df*	Mean Square	F	P value	ω^2
Arm	3.319	1	3.319	0.050	0.824	0.000
Residuals	4410.711	66	66.829			

3.7 PANAS Negative

Within Subjects Effects

Predictor	Sum of Squares	*df*	Mean Square	F	P value	ω^2
Time	499.18	1	499.18	24.019	$< .001$	0.053
Time × Arm	23.59	1	23.59	1.135	0.291	0.000
Residuals	1371.67	66	20.78			

Between Subjects Effects

Predictor	Sum of Squares	*df*	Mean Square	F	P value	ω^2
Arm	127.6	1	127.6	1.198	0.278	0.001
Residuals	7030.0	66	106.5			

3.8 R-UCLA

Within Subjects Effects

Predictor	Sum of Squares	*df*	Mean Square	F	P value	ω^2
Time	82.41	1	82.41	3.466	0.067	0.003
Time × Arm	31.15	1	31.15	1.310	0.257	0.000
Residuals	1569.32	66	23.78			

Between Subjects Effects

Predictor	Sum of Squares	*df*	Mean Square	F	P value	ω^2
Arm	217.9	1	217.9	0.689	0.409	0.000
Residuals	20862.1	66	316.1			

4 Intervention stability: RM ANOVA with T1 (baseline) vs T3 (at a one month follow-up)

4.1 CESD-R

Within Subjects Effects

Predictor	Sum of Squares	*df*	Mean Square	*F*	*P* value	ω^2
Time	2809.38	1	2809.38	32.102	$< .001$	0.108
Time × Arm	40.02	1	40.02	0.457	0.502	0.000
Residuals	4988.23	57	87.51			

Between Subjects Effects

Predictor	Sum of Squares	*df*	Mean Square	*F*	*P* value	ω^2
Arm	343.3	1	343.3	1.139	0.290	0.001
Residuals	17178.1	57	301.4			

4.2 PHQ-9

Within Subjects Effects

Predictor	Sum of Squares	*df*	Mean Square	*F*	*P* value	ω^2
Time	232.628	1	232.628	26.580	$< .001$	0.067
Time × Arm	1.916	1	1.916	0.219	0.642	0.000
Residuals	498.863	57	8.752			

Between Subjects Effects

Predictor	Sum of Squares	*df*	Mean Square	*F*	*P* value	ω^2
Arm	119.7	1	119.74	2.659	0.109	0.014
Residuals	2567.3	57	45.04			

4.3 PSWQ

Within Subjects Effects

Predictor	Sum of Squares	*df*	Mean Square	*F*	*P* value	ω^2
Time	236.465	1	236.465	12.136	$< .001$	0.014
Time × Arm	8.194	1	8.194	0.421	0.519	0.000
Residuals	1110.586	57	19.484			

Between Subjects Effects

Predictor	Sum of Squares	*df*	Mean Square	*F*	*P* value	ω^2
Arm	306.5	1	306.5	1.301	0.259	0.003
Residuals	13431.2	57	235.6			

4.4 STAI

Within Subjects Effects

Predictor	Sum of Squares	*df*	Mean Square	*F*	*P* value	ω^2
Time	337.742	1	337.742	18.115	$< .001$	0.034
Time × Arm	2.148	1	2.148	0.115	0.736	0.000
Residuals	1062.750	57	18.645			

Between Subjects Effects

Predictor	Sum of Squares	*df*	Mean Square	*F*	*P* value	ω^2
Arm	96.59	1	96.59	0.707	0.404	0.000
Residuals	7791.77	57	136.70			

4.5 SWLS

Within Subjects Effects

Predictor	Sum of Squares	*df*	Mean Square	*F*	*P* value	ω^2
Time	52.521	1	52.521	7.222	0.009	0.009
Time × Arm	4.724	1	4.724	0.650	0.424	0.000
Residuals	414.530	57	7.272			

Between Subjects Effects

Predictor	Sum of Squares	*df*	Mean Square	*F*	*P* value	ω^2
Arm	2.776	1	2.776	0.037	0.848	0.000
Residuals	4246.343	57	74.497			

4.6 PANAS Positive

Within Subjects Effects

Predictor	Sum of Squares	*df*	Mean Square	*F*	*P* value	ω^2
Time	185.090	1	185.090	14.873	$< .001$	0.041
Time × Arm	2.040	1	2.040	0.164	0.687	0.000
Residuals	709.333	57	12.444			

Between Subjects Effects

Predictor	Sum of Squares	*df*	Mean Square	*F*	*P* value	ω^2
Arm	20.34	1	20.34	0.354	0.554	0.000
Residuals	3271.00	57	57.39			

4.7 PANAS Negative

Within Subjects Effects

Predictor	Sum of Squares	*df*	Mean Square	*F*	*P* value	ω^2
Time	561.929	1	561.929	21.666	$< .001$	0.062
Time × Arm	2.403	1	2.403	0.093	0.762	0.000
Residuals	1478.359	57	25.936			

Between Subjects Effects

Predictor	Sum of Squares	*df*	Mean Square	*F*	*P* value	ω^2
Arm	123.7	1	123.7	1.083	0.302	0.001
Residuals	6506.7	57	114.2			

4.8 R-UCLA

Within Subjects Effects

Predictor	Sum of Squares	*df*	Mean Square	*F*	*P* value	ω^2
Time	35.858	1	35.858	1.192	0.279	0.000
Time × Arm	100.942	1	100.942	3.356	0.072	0.004
Residuals	1714.481	57	30.079			

Between Subjects Effects

Predictor	Sum of Squares	*df*	Mean Square	*F*	*P* value	ω^2
Arm	0.791	1	0.791	0.003	0.959	0.000
Residuals	16762.565	57	294.080			
