# Supplementary material for: Effectiveness of a Web-based and Mobile Therapy Chatbot on Anxiety and Depressive Symptoms in Subclinical Young Adults: Randomized Controlled Trial
Source: JMIR Form Res. 2024 Mar 20;8:e47960. doi: 10.2196/47960 (PMC10993129; doi:10.2196/47960)
Supplement: Multimedia Appendix 6 [file formative_v8i1e47960_app6.pdf]

CONSORT-EHEALTH (V 1.6.1) - Submission/Publication Form

The CONSORT-EHEALTH checklist is intended for authors of randomized trials evaluating web-based and Internet-based applications/interventions, including mobile interventions, electronic games (incl multiplayer games), social media, certain telehealth applications, and other interactive and/or networked electronic applications. Some of the items (e.g. all subitems under item 5 - description of the intervention) may also be applicable for other study designs.

The goal of the CONSORT EHEALTH checklist and guideline is to be
a) a guide for reporting for authors of RCTs,
b) to form a basis for appraisal of an ehealth trial (in terms of validity)

CONSORT-EHEALTH items/subitems are MANDATORY reporting items for studies published in the Journal of Medical Internet Research and other journals / scientific societies endorsing the checklist.

Items numbered 1., 2., 3., 4a., 4b etc are original CONSORT or CONSORT-NPT (non-pharmacologic treatment) items.
Items with Roman numerals (i., ii, iii, iv etc.) are CONSORT-EHEALTH extensions/clarifications.

As the CONSORT-EHEALTH checklist is still considered in a formative stage, we would ask that you also RATE ON A SCALE OF 1-5 how important/useful you feel each item is FOR THE PURPOSE OF THE CHECKLIST and reporting guideline (optional).

Mandatory reporting items are marked with a red *.
In the textboxes, either copy & paste the relevant sections from your manuscript into this form - please include any quotes from your manuscript in QUOTATION MARKS,
or answer directly by providing additional information not in the manuscript, or elaborating on why the item was not relevant for this study.

YOUR ANSWERS WILL BE PUBLISHED AS A SUPPLEMENTARY FILE TO YOUR PUBLICATION IN JMIR AND ARE CONSIDERED PART OF YOUR PUBLICATION (IF ACCEPTED).
Please fill in these questions diligently. Information will not be copyedited, so please use proper spelling and grammar, use correct capitalization, and avoid abbreviations.

DO NOT FORGET TO SAVE AS PDF _AND_ CLICK THE SUBMIT BUTTON SO YOUR ANSWERS ARE IN OUR DATABASE !!!

Citation Suggestion (if you append the pdf as Appendix we suggest to cite this paper in the caption):
Eysenbach G, CONSORT-EHEALTH Group
CONSORT-EHEALTH: Improving and Standardizing Evaluation Reports of Web-based and Mobile Health Interventions
J Med Internet Res 2011;13(4):e126
URL: http://www.jmir.org/2011/4/e126/
doi: 10.2196/jmir.1923
PMID: 22209829

rszymanski@swps.edu.pl (nieudostępniony)
Przełącz konto

Wersja robocza została zapisana

*Wymagane

Your name *
First Last

Robert Szymański

Primary Affiliation (short), City, Country *
University of Toronto, Toronto, Canada

SWPS University, Warsaw, Poland

Your e-mail address *
abc@gmail.com

rszymanski@swps.edu.pl

Title of your manuscript *
Provide the (draft) title of your manuscript.

Effectiveness of a Web-based and Mobile Therapy Chatbot on Anxiety and Depressive Symptoms in Subclinical Young Adults: A Randomized Controlled Trial

Name of your App/Software/Intervention *
If there is a short and a long/alternate name, write the short name first and add the long name in brackets.

Fido Chatbot

Evaluated Version (if any)
e.g. "V1", "Release 2017-03-01", "Version 2.0.27913"

A pre-release version

Language(s) *
What language is the intervention/app in? If multiple languages are available, separate by comma (e.g. "English, French")

Polish

URL of your Intervention Website or App

e.g. a direct link to the mobile app on app in appstore (itunes, Google Play), or URL of the website. If the intervention is a DVD or hardware, you can also link to an Amazon page.

https://fido.aid.pl

URL of an image/screenshot (optional)

Twoja odpowiedź

Accessibility *

Can an enduser access the intervention presently?

- access is free and open
- access only for special usergroups, not open
- access is open to everyone, but requires payment/subscription/in-app purchases
- app/intervention no longer accessible
- (selected) Inne: For now, Fido is available only for special usergroups (study participant

Primary Medical Indication/Disease/Condition *

e.g. "Stress", "Diabetes", or define the target group in brackets after the condition, e.g. "Autism (Parents of children with)", "Alzheimers (Informal Caregivers of)"

Anxiety, Depression

Primary Outcomes measured in trial *

comma-separated list of primary outcomes reported in the trial

depression symptoms, worry tendency, anxiety

Secondary/other outcomes

Secondary/other outcomes
Are there any other outcomes the intervention is expected to affect?

therapeutic alliance, user experience

Recommended "Dose" *
What do the instructions for users say on how often the app should be used?

- (x) Approximately Daily
- ( ) Approximately Weekly
- ( ) Approximately Monthly
- ( ) Approximately Yearly
- ( ) "as needed"
- ( ) Inne:

Approx. Percentage of Users (starters) still using the app as recommended after 3 months *

- (x) unknown / not evaluated
- ( ) 0-10%
- ( ) 11-20%
- ( ) 21-30%
- ( ) 31-40%
- ( ) 41-50%
- ( ) 51-60%
- ( ) 61-70%
- ( ) 71%-80%
- ( ) 81-90%
- ( ) 91-100%
- ( ) Inne:

Overall, was the app/intervention effective? *

- ○ yes: all primary outcomes were significantly better in intervention group vs control
- ○ partly: SOME primary outcomes were significantly better in intervention group vs control
- ◉ no statistically significant difference between control and intervention
- ○ potentially harmful: control was significantly better than intervention in one or more outcomes
- ○ inconclusive: more research is needed
- ○ Inne:

Article Preparation Status/Stage *

At which stage in your article preparation are you currently (at the time you fill in this form)

- ○ not submitted yet - in early draft status
- ◉ not submitted yet - in late draft status, just before submission
- ○ submitted to a journal but not reviewed yet
- ○ submitted to a journal and after receiving initial reviewer comments
- ○ submitted to a journal and accepted, but not published yet
- ○ published
- ○ Inne:

Journal *

If you already know where you will submit this paper (or if it is already submitted), please provide the journal name (if it is not JMIR, provide the journal name under "other")

- ◉ not submitted yet / unclear where I will submit this
- ○ Journal of Medical Internet Research (JMIR)
- ○ JMIR mHealth and UHealth

- ( ) JMIR Serious Games
- ( ) JMIR Mental Health
- ( ) JMIR Public Health
- ( ) JMIR Formative Research
- ( ) Other JMIR sister journal
- ( ) Inne:

Is this a full powered effectiveness trial or a pilot/feasibility trial? *

- (x) Pilot/feasibility
- ( ) Fully powered

Manuscript tracking number *

If this is a JMIR submission, please provide the manuscript tracking number under "other" (The ms tracking number can be found in the submission acknowledgement email, or when you login as author in JMIR. If the paper is already published in JMIR, then the ms tracking number is the four-digit number at the end of the DOI, to be found at the bottom of each published article in JMIR)

- (x) no ms number (yet) / not (yet) submitted to / published in JMIR
- ( ) Inne:

TITLE AND ABSTRACT

1a) TITLE: Identification as a randomized trial in the title

1a) Does your paper address CONSORT item 1a? *

I.e does the title contain the phrase "Randomized Controlled Trial"? (if not, explain the reason under "other")

- (x) yes
- ( ) Inne:

1a-i) Identify the mode of delivery in the title

Identify the mode of delivery. Preferably use “web-based” and/or “mobile” and/or “electronic game” in the title. Avoid ambiguous terms like “online”, “virtual”, “interactive”

"electronic game" in the title. Avoid ambiguous terms like "online", "virtual", "interactive". Use "Internet-based" only if Intervention includes non-web-based Internet components (e.g. email), use "computer-based" or "electronic" only if offline products are used. Use "virtual" only in the context of "virtual reality" (3-D worlds). Use "online" only in the context of "online support groups". Complement or substitute product names with broader terms for the class of products (such as "mobile" or "smart phone" instead of "iphone"), especially if the application runs on different platforms.

	1	2	3	4	5	
subitem not at all important	○	○	○	○	○	essential

Does your paper address subitem 1a-i? *

Copy and paste relevant sections from manuscript title (include quotes in quotation marks "like this" to indicate direct quotes from your manuscript), or elaborate on this item by providing additional information not in the ms, or briefly explain why the item is not applicable/relevant for your study

Yes, like this:
Effectiveness of a Web-based and Mobile Therapy Chatbot on Anxiety and Depressive Symptoms in Subclinical Young Adults: A Randomized Controlled Trial

1a-ii) Non-web-based components or important co-interventions in title

Mention non-web-based components or important co-interventions in title, if any (e.g., "with telephone support").

	1	2	3	4	5	
subitem not at all important	○	○	○	○	○	essential

Does your paper address subitem 1a-ii?

Copy and paste relevant sections from manuscript title (include quotes in quotation marks "like this" to indicate direct quotes from your manuscript), or elaborate on this item by providing additional information not in the ms, or briefly explain why the item is not applicable/relevant for your study

This item is not relevant, the intervention didn't include non-web-based components.

1a-iii) Primary condition or target group in the title

Mention primary condition or target group in the title, if any (e.g., “for children with Type I Diabetes”) Example: A Web-based and Mobile Intervention with Telephone Support for Children with Type I Diabetes: Randomized Controlled Trial

	1	2	3	4	5	
subitem not at all important	○	○	○	○	○	essential

Does your paper address subitem 1a-iii? *

Copy and paste relevant sections from manuscript title (include quotes in quotation marks "like this" to indicate direct quotes from your manuscript), or elaborate on this item by providing additional information not in the ms, or briefly explain why the item is not applicable/relevant for your study

Yes, like this:
Anxiety and Depressive Symptoms in Subclinical Young Adults

1b) ABSTRACT: Structured summary of trial design, methods, results, and conclusions

NPT extension: Description of experimental treatment, comparator, care providers, centers, and blinding status.

1b-i) Key features/functionalities/components of the intervention and comparator in the METHODS section of the ABSTRACT

Mention key features/functionalities/components of the intervention and comparator in the abstract. If possible, also mention theories and principles used for designing the site. Keep in mind the needs of systematic reviewers and indexers by including important synonyms. (Note: Only report in the abstract what the main paper is reporting. If this information is missing from the main body of text, consider adding it)

	1	2	3	4	5	
subitem not at all important	○	○	○	○	○	essential

Does your paper address subitem 1b-i? *

Copy and paste relevant sections from the manuscript abstract (include quotes in quotation marks "like this" to indicate direct quotes from your manuscript), or elaborate on this item by providing additional information not in the ms, or briefly explain why the item is not applicable/relevant for your study

Yes, like this:
We conducted a two-arm, open-label RCT on 81 participants with subclinical depression or anxiety recruited via social media. Participants were divided into two groups: experimental (that received a fully-automated Fido chatbot) and control (that received a self-help book). Both intervention methods addressed topics such as general psychoeducation and cognitive distortions identification and modification via socratic questioning. The chatbot also featured suicidal ideation identification and redirection to suicide hotlines.

1b-ii) Level of human involvement in the METHODS section of the ABSTRACT

Clarify the level of human involvement in the abstract, e.g., use phrases like “fully automated” vs. “therapist/nurse/care provider/physician-assisted” (mention number and expertise of providers involved, if any). (Note: Only report in the abstract what the main paper is reporting. If this information is missing from the main body of text, consider adding it)

	1	2	3	4	5	
subitem not at all important	○	○	○	○	○	essential

Does your paper address subitem 1b-ii?

Copy and paste relevant sections from the manuscript abstract (include quotes in quotation marks "like this" to indicate direct quotes from your manuscript), or elaborate on this item by providing additional information not in the ms, or briefly explain why the item is not applicable/relevant for your study

Yes, like this:
a fully-automated Fido chatbot

1b-iii) Open vs. closed, web-based (self-assessment) vs. face-to-face assessments in the METHODS section of the ABSTRACT

Mention how participants were recruited (online vs. offline), e.g., from an open access website or from a clinic or a closed online user group (closed usergroup trial), and clarify if this was a purely web-based trial, or there were face-to-face components (as part of the intervention or for assessment). Clearly say if outcomes were self-assessed through questionnaires (as common in web-based trials). Note: In traditional offline trials, an open trial (open-label trial) is a type of clinical trial in which both the researchers and participants know which treatment is being administered. To avoid confusion, use “blinded” or “unblinded” to indicated the level of blinding instead of “open”, as “open” in

web-based trials usually refers to “open access” (i.e. participants can self-enrol). (Note: Only report in the abstract what the main paper is reporting. If this information is missing from the main body of text, consider adding it)

	1	2	3	4	5	
subitem not at all important	○	○	○	○	○	essential

Does your paper address subitem 1b-iii?

Copy and paste relevant sections from the manuscript abstract (include quotes in quotation marks "like this" to indicate direct quotes from your manuscript), or elaborate on this item by providing additional information not in the ms, or briefly explain why the item is not applicable/relevant for your study

Yes, like this:
We used self-assessment scales to measure depression, anxiety, worry tendencies, satisfaction with life and loneliness at baseline, after 2 weeks intervention and at 1-month follow-up. We also controlled engagement and frequency of usage.

1b-iv) RESULTS section in abstract must contain use data

Report number of participants enrolled/assessed in each group, the use/uptake of the intervention (e.g., attrition/adherence metrics, use over time, number of logins etc.), in addition to primary/secondary outcomes. (Note: Only report in the abstract what the main paper is reporting. If this information is missing from the main body of text, consider adding it)

	1	2	3	4	5	
subitem not at all important	○	○	○	○	○	essential

Does your paper address subitem 1b-iv?

Copy and paste relevant sections from the manuscript abstract (include quotes in quotation marks "like this" to indicate direct quotes from your manuscript), or elaborate on this item by providing additional information not in the ms, or briefly explain why the item is not applicable/relevant for your study

Yes, like this:

"After intervention, depressive and anxiety symptoms were reduced in both groups (chatbot: N=36, control: N=38), which remained stable at 1-month follow-up. There was no loneliness difference after intervention in both groups, however in-depth exploratory analysis provided evidence that there was a decline among participants that had been using Fido more frequently. Both groups used their intervention technique with similar frequency, however the control group (mean 117.57 minutes, SD 72.40) spent more time on the intervention than the Fido group (mean 79.44 minutes, SD 42.96)."

1b-v) CONCLUSIONS/DISCUSSION in abstract for negative trials

Conclusions/Discussions in abstract for negative trials: Discuss the primary outcome - if the trial is negative (primary outcome not changed), and the intervention was not used, discuss whether negative results are attributable to lack of uptake and discuss reasons. (Note: Only report in the abstract what the main paper is reporting. If this information is missing from the main body of text, consider adding it)

	1	2	3	4	5	
subitem not at all important	○	○	○	○	○	essential

Does your paper address subitem 1b-v?

Copy and paste relevant sections from the manuscript abstract (include quotes in quotation marks "like this" to indicate direct quotes from your manuscript), or elaborate on this item by providing additional information not in the ms, or briefly explain why the item is not applicable/relevant for your study

Item not relevant, the trial was generally positive.

INTRODUCTION

2a) In INTRODUCTION: Scientific background and explanation of rationale

2a-i) Problem and the type of system/solution

Describe the problem and the type of system/solution that is object of the study: intended as stand-alone intervention vs. incorporated in broader health care program? Intended for a particular patient population? Goals of the intervention, e.g., being more cost-effective to other interventions, replace or complement other solutions? (Note: Details about the intervention are provided in “Methods” under 5)

	1	2	3	4	5	
subitem not at all important	○	○	○	○	○	essential

Does your paper address subitem 2a-i? *

Copy and paste relevant sections from the manuscript (include quotes in quotation marks "like this" to indicate direct quotes from your manuscript), or elaborate on this item by providing additional information not in the ms, or briefly explain why the item is not applicable/relevant for your study

Yes, like this:
"Therapy applications have been proven to be helpful for clients not able to afford to attend traditional therapy and for therapists seeking solutions to increase client engagement in therapy [2]."

2a-ii) Scientific background, rationale: What is known about the (type of) system

Scientific background, rationale: What is known about the (type of) system that is the object of the study (be sure to discuss the use of similar systems for other conditions/diagnoses, if appropiate), motivation for the study, i.e. what are the reasons for and what is the context for this specific study, from which stakeholder viewpoint is the study performed, potential impact of findings [2]. Briefly justify the choice of the comparator.

	1	2	3	4	5	
subitem not at all important	○	○	○	○	○	essential

Does your paper address subitem 2a-ii? *

Copy and paste relevant sections from the manuscript (include quotes in quotation marks "like this" to indicate direct quotes from your manuscript), or elaborate on this item by providing additional information not in the ms, or briefly explain why the item is not applicable/relevant for your study

Yes, like this:
"A recent meta-analysis [3] shows that using an internet-based intervention can be as effective as face-to-face therapy. Nevertheless, applications that are available on the market often lack appropriate scientific evidence of feasibility or efficacy [4]."
or
"In a randomized control trial (RCT) using Woebot for a period of 2 weeks has been proven to be more effective in reducing symptoms of anxiety and depression than the use of self-help materials prepared by WHO [6]. Further studies provided evidence that users can develop a bond with the Woebot on a similar level as the one that is built between the client and the therapist during a group-CBT [5,7]. Previous experiences with English-speaking mental health care chatbots (eg. Woebot, Wysa, Youper [8–10]) encourage the attempts to develop chatbots in other language versions, German [11], Chinese [12], Spanish [13], Ukrainian [14] or even multilingual [15]. Currently, the development of such applications varies among developed and developing countries, due to cross-cultural differences and specific obstacles [16]. In Poland, there is still a limited number of any digital therapeutics solutions, although the need for them is growing, as in the last few years there was a visible decline in mental health among Polish teenagers and young adults, which became especially severe during the COVID-19 pandemic [17]."

2b) In INTRODUCTION: Specific objectives or hypotheses

Does your paper address CONSORT subitem 2b? *

Copy and paste relevant sections from the manuscript (include quotes in quotation marks "like this" to indicate direct quotes from your manuscript), or elaborate on this item by providing additional information not in the ms, or briefly explain why the item is not applicable/relevant for your study

Yes, like this:
"Previous study on human-chatbot interaction using Fido provided satisfactory results, suggesting that Fido is pleasant in use, however, it hasn't been optimal yet and required further development in the area of user experience [18]. After its UX optimization Fido has

[illegible]
been ready to be used for an efficacy study. In line, we performed the first RCT aimed at testing the effects of using Fido to reduce subclinical depression and anxiety symptoms and comparing them to the use of self-help materials from the book “Mind over mood” [19].

Based on previous clinical research of chatbots [6,8–10], we aimed to investigate direct intervention effects and their stability. We hypothesized, that after two weeks of the intervention and after a 1-month follow-up:
1. The chatbot group will report lower depression and anxiety/worry symptoms than those with the self-help book only.
2. The chatbot group will have higher satisfaction with life than those using the self-help book only.
3. The positive affect will be higher, and the negative affect will be lower than prior to intervention. Furthermore, this change will be greater in the chatbot group than in the group with the self-help book.

Based on previous research on the chatbot-user interaction [8,18] we also hypothesized that:
4. The loneliness will be lower in the chatbot group than in the group with the self-help book.
5. Participants will form a bond with the chatbot, scoring at least 4 on the Working Alliance Inventory-Short Revised scale.
6. User's assessment of chatbot's linguistic pragmatics will correlate positively with the level of user experience."

METHODS

3a) Description of trial design (such as parallel, factorial) including allocation ratio

Does your paper address CONSORT subitem 3a? *

Copy and paste relevant sections from the manuscript (include quotes in quotation marks "like this" to indicate direct quotes from your manuscript), or elaborate on this item by providing additional information not in the ms, or briefly explain why the item is not applicable/relevant for your study

Yes, like this:
"We employed a 2 x 3 mixed factorial design with two intervention arms (Fido chatbot vs self-help book) and three-time points (before the intervention, after the intervention, and at a follow-up one month after the previous measurement). The primary intervention lasted two weeks. After the intervention, the use of the technique was not obligatory (but it was not forbidden)."

3b) Important changes to methods after trial commencement (such as eligibility criteria), with reasons

Does your paper address CONSORT subitem 3b? *

Copy and paste relevant sections from the manuscript (include quotes in quotation marks "like this" to indicate direct quotes from your manuscript), or elaborate on this item by providing additional information not in the ms, or briefly explain why the item is not applicable/relevant for your study

Not relevant. We didn't have any important changes to methods.

3b-i) Bug fixes, Downtimes, Content Changes

Bug fixes, Downtimes, Content Changes: ehealth systems are often dynamic systems. A description of changes to methods therefore also includes important changes made on the intervention or comparator during the trial (e.g., major bug fixes or changes in the functionality or content) (5-iii) and other "unexpected events" that may have influenced study design such as staff changes, system failures/downtimes, etc. [2].

	1	2	3	4	5	
subitem not at all important	○	○	○	○	○	essential

Does your paper address subitem 3b-i?

Copy and paste relevant sections from the manuscript (include quotes in quotation marks "like this" to indicate direct quotes from your manuscript), or elaborate on this item by providing additional information not in the ms, or briefly explain why the item is not applicable/relevant for your study

Yes, like this:
"During the trial none of the features underwent any changes."

4a) Eligibility criteria for participants

Does your paper address CONSORT subitem 4a? *

Copy and paste relevant sections from the manuscript (include quotes in quotation marks "like this" to indicate direct quotes from your manuscript), or elaborate on this item by providing additional information not in the ms, or briefly explain why the item is not applicable/relevant for your study

Yes, like this:
"Eligibility criteria included: (a) age between 18 and 35 years, (b) not undergoing psychotherapy, coaching or psychopharmacological treatment, (c) not being diagnosed with a neurological disorder, (d) declaring at least mild depressive or anxiety symptoms by achieving a total score of at least 16 points on Center for Epidemiologic Studies Depression Scale Revised (CESD-R) [20,21] or at least 50 points on Penn State Worry Questionnaire (PSWQ) [22,23], (e) being able to visit the study site in Poznań (Poland) to complete follow-up measurements. Computer literacy and proficiency in Polish were implicitly presumed as all participants filled in an online screening survey written in Polish."

4a-i) Computer / Internet literacy

Computer / Internet literacy is often an implicit “de facto” eligibility criterion - this should be explicitly clarified.

	1	2	3	4	5	
subitem not at all important	○	○	○	○	○	essential

Does your paper address subitem 4a-i?

Copy and paste relevant sections from the manuscript (include quotes in quotation marks "like this" to indicate direct quotes from your manuscript), or elaborate on this item by providing additional information not in the ms, or briefly explain why the item is not applicable/relevant for your study

Yes, like this:
"Computer literacy and proficiency in Polish were implicitly presumed as all participants filled in an online screening survey written in Polish."

4a-ii) Open vs. closed, web-based vs. face-to-face assessments:

Open vs. closed, web-based vs. face-to-face assessments: Mention how participants were recruited (online vs. offline), e.g., from an open access website or from a clinic, and clarify if this was a purely web-based trial, or there were face-to-face components (as part of the intervention or for assessment), i.e., to what degree got the study team to know the participant. In online-only trials, clarify if participants were quasi-anonymous and whether having multiple identities was possible or whether technical or logistical measures (e.g., cookies, email confirmation, phone calls) were used to detect/prevent these.

	1	2	3	4	5	
subitem not at all important	○	○	○	○	○	essential

Does your paper address subitem 4a-ii? *

Copy and paste relevant sections from the manuscript (include quotes in quotation marks "like this" to indicate direct quotes from your manuscript), or elaborate on this item by providing additional information not in the ms, or briefly explain why the item is not applicable/relevant for your study

Yes, like this:
"Participants were recruited via Facebook and Instagram advertisements from May to June 2022 and screened using a Qualtrics online survey (measurement at T0). The estimated sample size was 80 participants with an estimated dropout of around 15%."
and
"All questionnaires were implemented in Qualtrics and administered mostly offline, in the study site; when participants could not visit the lab, they were given a link to an online version of the survey"

4a-iii) Information giving during recruitment

Information given during recruitment. Specify how participants were briefed for recruitment and in the informed consent procedures (e.g., publish the informed consent documentation as appendix, see also item X26), as this information may have an effect on user self-selection, user expectation and may also bias results.

	1	2	3	4	5	
subitem not at all important	○	○	○	○	○	essential

Does your paper address subitem 4a-iii?

Copy and paste relevant sections from the manuscript (include quotes in quotation marks "like this" to indicate direct quotes from your manuscript), or elaborate on this item by providing additional information not in the ms, or briefly explain why the item is not applicable/relevant for your study

Yes, like this:
"Because the intervention involved either using the chatbot or the book, participants were informed about their assignment. To reduce the effect of expectation bias, we intentionally masked our hypotheses about the superiority of chatbot-supported therapy

masked our hypotheses about the superiority of chatbot supported therapy.

During the onboarding for the intervention, the participants were asked to use the assigned intervention technique as needed i.e. with no pre-specified minimal time of use per day or week. The uptake of the intervention was monitored via regular commitment checks delivered by email, which could serve as a reminder to use the assigned intervention technique."

4b) Settings and locations where the data were collected

Does your paper address CONSORT subitem 4b? *

Copy and paste relevant sections from the manuscript (include quotes in quotation marks "like this" to indicate direct quotes from your manuscript), or elaborate on this item by providing additional information not in the ms, or briefly explain why the item is not applicable/relevant for your study

Yes, like this:
"All questionnaires were implemented in Qualtrics and administered mostly offline, in the study site; when participants could not visit the lab, they were given a link to an online version of the survey."

4b-i) Report if outcomes were (self-)assessed through online questionnaires

Clearly report if outcomes were (self-)assessed through online questionnaires (as common in web-based trials) or otherwise.

	1	2	3	4	5	
subitem not at all important	○	○	○	○	○	essential

Does your paper address subitem 4b-i? *

Copy and paste relevant sections from the manuscript (include quotes in quotation marks "like this" to indicate direct quotes from your manuscript), or elaborate on this item by providing additional information not in the ms, or briefly explain why the item is not applicable/relevant for your study

Yes, like this:
"Primary outcomes were assessed at three time points: baseline (T1), after two weeks of the intervention (T2) and at a follow-up one month after the previous measurement (T3). All questionnaires were implemented in Qualtrics and administered mostly offline, in the study

[illegible]
site; when participants could not visit the lab, they were given a link to an online version of the survey. Participants filled in validated Polish adaptations of the scales. For primary analyses we used sum scores for every single primary outcome measured at one time point (not change scores)."

4b-ii) Report how institutional affiliations are displayed

Report how institutional affiliations are displayed to potential participants [on ehealth media], as affiliations with prestigious hospitals or universities may affect volunteer rates, use, and reactions with regards to an intervention.(Not a required item – describe only if this may bias results)

	1	2	3	4	5	
subitem not at all important	○	○	○	○	○	essential

Does your paper address subitem 4b-ii?

Copy and paste relevant sections from the manuscript (include quotes in quotation marks "like this" to indicate direct quotes from your manuscript), or elaborate on this item by providing additional information not in the ms, or briefly explain why the item is not applicable/relevant for your study

Not relevant. We used the logo of our university.

5) The interventions for each group with sufficient details to allow replication, including how and when they were actually administered

5-i) Mention names, credential, affiliations of the developers, sponsors, and owners

Mention names, credential, affiliations of the developers, sponsors, and owners [6] (if authors/evaluators are owners or developer of the software, this needs to be declared in a “Conflict of interest” section or mentioned elsewhere in the manuscript).

	1	2	3	4	5	
subitem not at all important	○	○	○	○	○	essential

Does your paper address subitem 5-i?

Copy and paste relevant sections from the manuscript (include quotes in quotation marks "like this" to indicate direct quotes from your manuscript), or elaborate on this item by providing additional information not in the ms, or briefly explain why the item is
[illegible]

not applicable/relevant for your study

Yes, like this:
"All authors are actively involved in the development of Fido at a pre-release stage yet declare no financial benefits from this project other than from a publicly funded grant mentioned above."

5-ii) Describe the history/development process

Describe the history/development process of the application and previous formative evaluations (e.g., focus groups, usability testing), as these will have an impact on adoption/use rates and help with interpreting results.

	1	2	3	4	5	
subitem not at all important	○	○	○	○	○	essential

Does your paper address subitem 5-ii?

Copy and paste relevant sections from the manuscript (include quotes in quotation marks "like this" to indicate direct quotes from your manuscript), or elaborate on this item by providing additional information not in the ms, or briefly explain why the item is not applicable/relevant for your study

Yes, like this:
"It’s been developed using iterative co-development with focus groups consisting of therapists and potential users (for more information, see [18]). Moreover, external therapists provided quality assurance for all therapeutic methods used by the chatbot, while software engineers used standard testing procedures."

5-iii) Revisions and updating

Revisions and updating. Clearly mention the date and/or version number of the application/intervention (and comparator, if applicable) evaluated, or describe whether the intervention underwent major changes during the evaluation process, or whether the development and/or content was “frozen” during the trial. Describe dynamic components such as news feeds or changing content which may have an impact on the replicability of the intervention (for unexpected events see item 3b).

	1	2	3	4	5	
subitem not at all important	○	○	○	○	○	essential

Does your paper address subitem 5-iii?

Copy and paste relevant sections from the manuscript (include quotes in quotation marks "like this" to indicate direct quotes from your manuscript), or elaborate on this item by providing additional information not in the ms, or briefly explain why the item is not applicable/relevant for your study

Yes, like this:
"During the trial none of the features underwent any changes."

5-iv) Quality assurance methods

Provide information on quality assurance methods to ensure accuracy and quality of information provided [1], if applicable.

	1	2	3	4	5	
subitem not at all important	○	○	○	○	○	essential

Does your paper address subitem 5-iv?

Copy and paste relevant sections from the manuscript (include quotes in quotation marks "like this" to indicate direct quotes from your manuscript), or elaborate on this item by providing additional information not in the ms, or briefly explain why the item is not applicable/relevant for your study

Yes, like this:
"Moreover, external therapists provided quality assurance for all therapeutic methods used by the chatbot, while software engineers used standard testing procedures."

5-v) Ensure replicability by publishing the source code, and/or providing screenshots/screen-capture video, and/or providing flowcharts of the algorithms used

Ensure replicability by publishing the source code, and/or providing screenshots/screen-capture video, and/or providing flowcharts of the algorithms used. Replicability (i.e., other researchers should in principle be able to replicate the study) is a hallmark of scientific reporting.

1 2 3 4 5

subitem not at all important	○	○	○	○	○	essential

Does your paper address subitem 5-v?

Copy and paste relevant sections from the manuscript (include quotes in quotation marks "like this" to indicate direct quotes from your manuscript), or elaborate on this item by providing additional information not in the ms, or briefly explain why the item is not applicable/relevant for your study

Publishing the source code is not possible. However, the chatbot will be publicly and freely available from the Fido website (https://fido.aid.pl). We included a screenshot of a sample user-chatbot conversation (translated to English) in the manuscript.

5-vi) Digital preservation

Digital preservation: Provide the URL of the application, but as the intervention is likely to change or disappear over the course of the years; also make sure the intervention is archived (Internet Archive, webcitation.org, and/or publishing the source code or screenshots/videos alongside the article). As pages behind login screens cannot be archived, consider creating demo pages which are accessible without login.

	1	2	3	4	5	
subitem not at all important	○	○	○	○	○	essential

Does your paper address subitem 5-vi?

Copy and paste relevant sections from the manuscript (include quotes in quotation marks "like this" to indicate direct quotes from your manuscript), or elaborate on this item by providing additional information not in the ms, or briefly explain why the item is not applicable/relevant for your study

URL of the application: https://fido.aid.pl

5-vii) Access

Access: Describe how participants accessed the application, in what setting/context, if they had to pay (or were paid) or not, whether they had to be a member of specific group. If known, describe how participants obtained “access to the platform and Internet” [1]. To ensure access for editors/reviewers/readers, consider to provide a “backdoor” login account or demo mode for reviewers/readers to explore the application (also important for archiving purposes, see vi).

1	2	3	4	5

subitem not at all important	◯	◯	◯	◯	◯	essential

Does your paper address subitem 5-vii? *

Copy and paste relevant sections from the manuscript (include quotes in quotation marks "like this" to indicate direct quotes from your manuscript), or elaborate on this item by providing additional information not in the ms, or briefly explain why the item is not applicable/relevant for your study

Yes, like this:
"For the experimental group we used a free, pre-release version of a therapy-supporting Polish-language chatbot Fido [26] integrated into Facebook Messenger. Participants were added as testers in Meta's development website and accessed the chatbot via links embedded in individual emails. "

5-viii) Mode of delivery, features/functionalities/components of the intervention and comparator, and the theoretical framework

Describe mode of delivery, features/functionalities/components of the intervention and comparator, and the theoretical framework [6] used to design them (instructional strategy [1], behaviour change techniques, persuasive features, etc., see e.g., [7, 8] for terminology). This includes an in-depth description of the content (including where it is coming from and who developed it) [1]," whether [and how] it is tailored to individual circumstances and allows users to track their progress and receive feedback" [6]. This also includes a description of communication delivery channels and – if computer-mediated communication is a component – whether communication was synchronous or asynchronous [6]. It also includes information on presentation strategies [1], including page design principles, average amount of text on pages, presence of hyperlinks to other resources, etc. [1].

	1	2	3	4	5	
subitem not at all important	◯	◯	◯	◯	◯	essential

Does your paper address subitem 5-viii? *

Copy and paste relevant sections from the manuscript (include quotes in quotation marks "like this" to indicate direct quotes from your manuscript), or elaborate on this item by providing additional information not in the ms, or briefly explain why the item is not applicable/relevant for your study

Party. Not as detailled, but we describe the chatbot like this:

"For the experimental group we used a free, pre-release version of a therapy-supporting Polish-language chatbot Fido [26] integrated into Facebook Messenger. Participants were added as testers in Meta's development website and accessed the chatbot via links embedded in individual emails. They were given no special training (apart from the initial e-mail instructions), but were offered technical assistance in case of any problems.

The chatbot uses machine learning models for intelligent user intent detection and close-ended input methods (such as choosing one option from a list). It's been developed using iterative co-development with focus groups consisting of therapists and potential users (for more information, see [18]). Moreover, external therapists provided quality assurance for all therapeutic methods used by the chatbot, while software engineers used standard testing procedures. During the trial none of the features underwent any changes.

During the onboarding procedure, participants indicate their gender and receive information on user terms and data protection, as well as basic training in cognitive biases because understanding them is crucial for interaction with Fido. After onboarding, users can try different therapeutic techniques implemented in Fido using a tree-based structure.

The primary functionality of Fido is providing a dialogue focused on intelligent recognition of cognitive biases and their subsequent modification using socratic questioning. It also recognizes suicidal ideation and reacts to it by redirecting users to emergency hotlines.

To maximize Fido's performance, one of its machine learning models implements the so-called ABC technique (known from CBT) which helps patients organize and differentiate between activating events, beliefs and their emotional or behavioral consequences [27], as presented in Figure 2. Fido also provides psychoeducation about depression, anxiety, and emotions. Lastly, it embraces gratitude practice exercises [19].
"
and for the control group:
"The chapters 1-6 and 12 from the Polish translation of "Mind over mood" [19,28] were used to familiarize control participants with similar contents and tasks as those provided in the experimental group. The book contains psychoeducation content with self-help exercises and is a well-established therapist's guide to depression. "

5-ix) Describe use parameters

Describe use parameters (e.g., intended “doses” and optimal timing for use). Clarify what instructions or recommendations were given to the user, e.g., regarding timing, frequency, heaviness of use, if any, or was the intervention used ad libitum.

	1	2	3	4	5	
subitem not at all important	○	○	○	○	○	essential

Does your paper address subitem 5-ix?

Copy and paste relevant sections from the manuscript (include quotes in quotation marks "like this" to indicate direct quotes from your manuscript), or elaborate on this item by providing additional information not in the ms, or briefly explain why the item is not applicable/relevant for your study

Yes, like this:
"During the onboarding for the intervention, the participants were asked to use the assigned intervention technique as needed i.e. with no pre-specified minimal time of use per day or week."

5-x) Clarify the level of human involvement

Clarify the level of human involvement (care providers or health professionals, also technical assistance) in the e-intervention or as co-intervention (detail number and expertise of professionals involved, if any, as well as “type of assistance offered, the timing and frequency of the support, how it is initiated, and the medium by which the assistance is delivered”. It may be necessary to distinguish between the level of human involvement required for the trial, and the level of human involvement required for a routine application outside of a RCT setting (discuss under item 21 – generalizability).

	1	2	3	4	5	
subitem not at all important	○	○	○	○	○	essential

Does your paper address subitem 5-x?

Copy and paste relevant sections from the manuscript (include quotes in quotation marks "like this" to indicate direct quotes from your manuscript), or elaborate on this item by providing additional information not in the ms, or briefly explain why the item is not applicable/relevant for your study

Yes, like this:
"They were given no special training (apart from the initial e-mail instructions), but were offered technical assistance in case of any problems."

5-xi) Report any prompts/reminders used

Report any prompts/reminders used: Clarify if there were prompts (letters, emails, phone calls, SMS) to use the application, what triggered them, frequency etc. It may be necessary to distinguish between the level of prompts/reminders required for the trial, and the level of prompts/reminders for a routine application outside of a RCT setting (discuss under item 21 – generalizability).

	1	2	3	4	5	
subitem not at all important	○	○	○	○	○	essential

Does your paper address subitem 5-xi? *

Copy and paste relevant sections from the manuscript (include quotes in quotation marks "like this" to indicate direct quotes from your manuscript), or elaborate on this item by providing additional information not in the ms, or briefly explain why the item is not applicable/relevant for your study

Yes, like this:
"The uptake of the intervention was monitored via regular commitment checks delivered by email, which could serve as a reminder to use the assigned intervention technique."

5-xii) Describe any co-interventions (incl. training/support)

Describe any co-interventions (incl. training/support): Clearly state any interventions that are provided in addition to the targeted eHealth intervention, as ehealth intervention may not be designed as stand-alone intervention. This includes training sessions and support [1]. It may be necessary to distinguish between the level of training required for the trial, and the level of training for a routine application outside of a RCT setting (discuss under item 21 – generalizability.

	1	2	3	4	5	
subitem not at all important	○	○	○	○	○	essential

Does your paper address subitem 5-xii? *

Copy and paste relevant sections from the manuscript (include quotes in quotation marks "like this" to indicate direct quotes from your manuscript), or elaborate on this item by providing additional information not in the ms, or briefly explain why the item is not applicable/relevant for your study

Not relevant, there were no co-interventions.

6a) Completely defined pre-specified primary and secondary outcome measures, including how and when they were assessed

Does your paper address CONSORT subitem 6a? *

Copy and paste relevant sections from the manuscript (include quotes in quotation marks "like this" to indicate direct quotes from your manuscript), or elaborate on this item by providing additional information not in the ms, or briefly explain why the item is not applicable/relevant for your study

Yes, like this:
"Primary outcomes were assessed at three time points: baseline (T1), after two weeks of the intervention (T2) and at a follow-up one month after the previous measurement (T3). All questionnaires were implemented in Qualtrics and administered mostly offline, in the study site; when participants could not visit the lab, they were given a link to an online version of the survey. Participants filled in validated Polish adaptations of the scales. For primary analyses we used sum scores for every single primary outcome measured at one time point (not change scores).

CESD-R
Depression symptom severity was measured using the Center for Epidemiologic Studies Depression Scale Revised [20,21]. It is a 20-item screening tool for a major depressive disorder. The scale uses nine symptom groups defined in DSM-5 [29]: sadness, anhedonia, appetite, sleep, thinking/concentration, guilt, fatigue, agitation and suicidal ideation.

PHQ-9
Another measure used to assess participants' depression was Patient Health Questionnaire-9 [30,31] from the self-administered version of PRIME-MD inventory [32]. This brief, 9-item scale is based on DSM-IV criteria for depression. It is used mainly for symptom severity monitoring in primary care.

PSWQ
We used Penn State Worry Questionnaire [22,23] to measure worry tendency, which is the primary component of generalized anxiety disorder. The 16-item scale addresses worry's excessiveness, generality, and uncontrollable dimensions.

STAI
State-Trait Anxiety Inventory [33,34] is another questionnaire used to measure anxiety, both as a temporary state and as a relatively fixed trait of an individual. We used a 20-item trait scale of STAI.

PANAS
Positive and Negative Affect Schedule [35,36] was used to measure the general affect during the last two weeks. Two subscale sum scores were calculated: one for the positive feelings (9 items) and another for the negative affect (9 items). The last two items of the original scale were not administered due to human error in an online survey implementation.

SWLS
Primary measures also included a brief, 5-item Satisfaction with Life Scale [37,38], which was used to assess global life satisfaction in a cognitive-judgemental aspect. Together with positive and negative affect, life satisfaction is an important component of subjective well-being.

R-UCLA

R-UCLA

We used the 20-item Revised UCLA Loneliness Scale [39,40] to assess other specific aspects of well-being, namely subjective feelings of loneliness and social isolation.

Secondary and other outcome measures

During the two-week intervention participants received five engagement check surveys, each containing a single question “How much time have you spent on therapeutic chatbot/book use during the last 48 hours?” with answers in 10-minute increments. We then calculated the sum score of the engagement checks as a total number of declared time spent on book/chatbot use in minutes. Each survey had to be completed in less than 24 hours.

After the intervention (T2) all participants also received an open-ended question “How many times have you used the chatbot / read the book in the past two weeks?”. If a participant declared a range of values (e.g. “10-12 times”), we recoded their answer to a median value in this range.

Another secondary measure used in both arms at T2 was the 12-item test which assessed the participants’ acquired knowledge of psychoeducation topics covered both by Fido and the “Mind over mood” book (see: Appendix 1). The test was administered in a paper-and-pencil format at the study site.

Participants from the chatbot arm also received several additional scales at T2:

WAI-SR

Working Alliance Inventory Short Revised [41] is a 12-item scale for measuring therapeutic alliance in three key areas defined by [42], which are: agreement on the Tasks (1) and Goals (2) of the therapy and an overall patient-therapist affective Bond (3). As agent-guided therapy also requires some form of therapeutic chatbot-user alliance, we adapted this scale to be used in our study. All items were first translated to Polish using a forward and backward translation with reconciliation. Next, we modified phrases related to human-led therapy to increase their relevance for the chatbot therapy (see: Appendix 2 for the original WAI-SR items and their adaptation for this study). Items from the Tasks and Goals subscales were not as relevant to this study (because Fido does not establish these elements of the intervention, they are determined ad hoc by the user), so alongside the total average scale score we also made use of the average of the Bond subscale.

UX

For the general assessment of user experience we included non-mandatory items from several scales used in other studies:

1) Acceptability E-scale [43] used to measure the overall acceptability and usability of health-related computer applications. The team prepared its Polish translation using a similar procedure to our adaptation of WAI-SR (see: Appendix 3 for both the original and the translation). The original scale included 5 items. One of the items regarded the functionality of the program. We used this item four times, as we wanted to check the functionality of four different techniques (psychoeducation, cognitive biases recognition, suicidal thoughts recognition, and gratitude practice). This way we ended up with nine items and used their sum as a total score.

2) Human-Chatbot Interaction Scale (HCIS) [44] with answers ranging from 1 to 7 (see: Appendix 4). Calculated sums from six subscales were used:

a) Supportive Anthropomorphic Traits (3 items),
b) Unsupportive Anthropomorphic Traits (4 items),
c) Behavioral Traits (7 items),
d) Uncanny Valley (6 items),
e) Competence (6 items),
f) Warmth (6 items).

3) Language Pragmaticality Scale (LPS) [18], which we previously used to assess the perceived ability of Fido to communicate in a pragmatically sound manner. The scale consists of four separately scored items with answers on a percentage scale (0–100%):

a) What percentage of overall chatbot statements were adequate?
b) What percentage of overall chatbot statements were neutral (neither adequate nor inadequate)?
c) What percentage of overall chatbot statements were inadequate?
d) In what percentage have you been feeling understood by a chatbot?

d) In what percentage have you been feeling understood by a chatbot?

The preregistered plan of the study also included chatbot metadata (frequency of use or time spent in the conversation) for each user after the intervention (T2). However, due to technical and ethical reasons, we could not link the questionnaire data with anonymous chatbot metadata. Therefore, we only used metadata (frequency of use) at a group level."

6a-i) Online questionnaires: describe if they were validated for online use and apply CHERRIES items to describe how the questionnaires were designed/deployed

If outcomes were obtained through online questionnaires, describe if they were validated for online use and apply CHERRIES items to describe how the questionnaires were designed/deployed [9].

	1	2	3	4	5	
subitem not at all important	○	○	○	○	○	essential

Does your paper address subitem 6a-i?

Copy and paste relevant sections from manuscript text

We did not use the CHERRIES items in the manuscript. The study mainly utilized commonly used, standardized, and validated questionnaires. Some of them have been validated for online use in English, but the Polish online versions still need to be validated. Based on the meta-analytic review by Gwaltney et al. (https://doi.org/10.1111/j.1524-4733.2007.00231.x), we believe there is extensive evidence that online and paper-and-pencil versions are equivalent.

6a-ii) Describe whether and how “use” (including intensity of use/dosage) was defined/measured/monitored

Describe whether and how “use” (including intensity of use/dosage) was defined/measured/monitored (logins, logfile analysis, etc.). Use/adoption metrics are important process outcomes that should be reported in any ehealth trial.

	1	2	3	4	5	
subitem not at all important	○	○	○	○	○	essential

Does your paper address subitem 6a-ii?

Copy and paste relevant sections from manuscript text

Yes, like this:
"During the two-week intervention participants received five engagement check surveys, each containing a single question “How much time have you spent on therapeutic chatbot/book use during the last 48 hours?” with answers in 10-minute increments. We then calculated the sum score of the engagement checks as a total number of declared time spent on book/chatbot use in minutes. Each survey had to be completed in less than 24

hours.

After the intervention (T2) all participants also received an open-ended question “How many times have you used the chatbot / read the book in the past two weeks?”. If a participant declared a range of values (e.g. “10-12 times”), we recoded their answer to a median value in this range."

6a-iii) Describe whether, how, and when qualitative feedback from participants was obtained

Describe whether, how, and when qualitative feedback from participants was obtained (e.g., through emails, feedback forms, interviews, focus groups).

	1	2	3	4	5	
subitem not at all important	○	○	○	○	○	essential

Does your paper address subitem 6a-iii?

Copy and paste relevant sections from manuscript text

Yes, like this:
"The team also collected informal, qualitative data through individual in-depth interviews and an open-ended survey question after the intervention (T2). Volunteers (N=16) from the chatbot group answered questions about their perceived advantages and pitfalls of Fido, as well as other unintended effects which had not been assessed by the previously administered questionnaires. "

6b) Any changes to trial outcomes after the trial commenced, with reasons

Does your paper address CONSORT subitem 6b? *

Copy and paste relevant sections from the manuscript (include quotes in quotation marks "like this" to indicate direct quotes from your manuscript), or elaborate on this item by providing additional information not in the ms, or briefly explain why the item is not applicable/relevant for your study

Yes, like this:
"The preregistered plan of the study also included chatbot metadata (frequency of use or time spent in the conversation) for each user after the intervention (T2). However, due to technical and ethical reasons, we could not link the questionnaire data with anonymous chatbot metadata. Therefore, we only used metadata (frequency of use) at a group level."

7a) How sample size was determined

NPT: When applicable, details of whether and how the clustering by care provides or centers was addressed

centers was addressed

7a-i) Describe whether and how expected attrition was taken into account when calculating the sample size

Describe whether and how expected attrition was taken into account when calculating the sample size.

	1	2	3	4	5	
subitem not at all important	○	○	○	○	○	essential

Does your paper address subitem 7a-i?

Copy and paste relevant sections from manuscript title (include quotes in quotation marks "like this" to indicate direct quotes from your manuscript), or elaborate on this item by providing additional information not in the ms, or briefly explain why the item is not applicable/relevant for your study

Yes, like this:
"The estimated sample size was 80 participants with an estimated dropout of around 15%."

7b) When applicable, explanation of any interim analyses and stopping guidelines

Does your paper address CONSORT subitem 7b? *

Copy and paste relevant sections from the manuscript (include quotes in quotation marks "like this" to indicate direct quotes from your manuscript), or elaborate on this item by providing additional information not in the ms, or briefly explain why the item is not applicable/relevant for your study

Not relevant, we didn't perform any intermin analyses.

8a) Method used to generate the random allocation sequence

NPT: When applicable, how care providers were allocated to each trial group

Does your paper address CONSORT subitem 8a? *

Copy and paste relevant sections from the manuscript (include quotes in quotation marks "like this" to indicate direct quotes from your manuscript), or elaborate on this item by providing additional information not in the ms, or briefly explain why the item is not applicable/relevant for your study

Yes, like this:
"After enrollment, simple randomization with a 1:1 ratio was performed using computer-generated random numbers (via Python script)."

8b) Type of randomisation; details of any restriction (such as blocking and block size)

Does your paper address CONSORT subitem 8b? *

Copy and paste relevant sections from the manuscript (include quotes in quotation marks "like this" to indicate direct quotes from your manuscript), or elaborate on this item by providing additional information not in the ms, or briefly explain why the item is not applicable/relevant for your study

Yes, like this:
"After enrollment, the research team members performed simple randomization with a 1:1 ratio (via Python script)."

9) Mechanism used to implement the random allocation sequence (such as sequentially numbered containers), describing any steps taken to conceal the sequence until interventions were assigned

Does your paper address CONSORT subitem 9? *

Copy and paste relevant sections from the manuscript (include quotes in quotation marks "like this" to indicate direct quotes from your manuscript), or elaborate on this item by providing additional information not in the ms, or briefly explain why the item is not applicable/relevant for your study

Yes, like this:
"After enrollment, the research team members performed simple randomization with a 1:1 ratio (via Python script)."

10) Who generated the random allocation sequence, who enrolled participants, and who assigned participants to interventions

Does your paper address CONSORT subitem 10? *

Copy and paste relevant sections from the manuscript (include quotes in quotation marks "like this" to indicate direct quotes from your manuscript), or elaborate on this item by providing additional information not in the ms, or briefly explain why the item is not applicable/relevant for your study

Yes, like this:
"After enrollment, the research team members performed simple randomization with a 1:1 ratio (via Python script)."

11a) If done, who was blinded after assignment to interventions (for example, participants, care providers, those assessing outcomes) and how

NPT: Whether or not administering co-interventions were blinded to group assignment

11a-i) Specify who was blinded, and who wasn't

Specify who was blinded, and who wasn't. Usually, in web-based trials it is not possible to blind the participants [1, 3] (this should be clearly acknowledged), but it may be possible to blind outcome assessors, those doing data analysis or those administering co-interventions (if any).

	1	2	3	4	5	
subitem not at all important	○	○	○	○	○	essential

Does your paper address subitem 11a-i? *

Copy and paste relevant sections from the manuscript (include quotes in quotation marks "like this" to indicate direct quotes from your manuscript), or elaborate on this item by providing additional information not in the ms, or briefly explain why the item is not applicable/relevant for your study

Yes, like this:
"Because the intervention involved either using the chatbot or the book, participants were informed about their assignment. To reduce the effect of expectation bias, we intentionally
[illegible]

masked our hypotheses about the superiority of chatbot-supported therapy."
and
"The research team members remained unblinded as well."

11a-ii) Discuss e.g., whether participants knew which intervention was the “intervention of interest” and which one was the “comparator”

Informed consent procedures (4a-ii) can create biases and certain expectations - discuss e.g., whether participants knew which intervention was the “intervention of interest” and which one was the “comparator”.

	1	2	3	4	5	
subitem not at all important	○	○	○	○	○	essential

Does your paper address subitem 11a-ii?

Copy and paste relevant sections from the manuscript (include quotes in quotation marks "like this" to indicate direct quotes from your manuscript), or elaborate on this item by providing additional information not in the ms, or briefly explain why the item is not applicable/relevant for your study

The participants didn't know which intervention is of main interest, and which one is the comparator. We mentioned it like this:
"To reduce the effect of expectation bias, we intentionally masked our hypotheses about the superiority of chatbot-supported therapy.""

11b) If relevant, description of the similarity of interventions

(this item is usually not relevant for ehealth trials as it refers to similarity of a placebo or sham intervention to a active medication/intervention)

Does your paper address CONSORT subitem 11b? *

Copy and paste relevant sections from the manuscript (include quotes in quotation marks "like this" to indicate direct quotes from your manuscript), or elaborate on this item by providing additional information not in the ms, or briefly explain why the item is not applicable/relevant for your study

This item is not relevant.

12a) Statistical methods used to compare groups for primary and secondary outcomes

NPT: When applicable, details of whether and how the clustering by care providers or centers was addressed

Does your paper address CONSORT subitem 12a? *

Copy and paste relevant sections from the manuscript (include quotes in quotation marks "like this" to indicate direct quotes from your manuscript), or elaborate on this item by providing additional information not in the ms, or briefly explain why the item is not applicable/relevant for your study

Yes, like this:
"For the baseline, we conducted contingency analyses on nominal and ordinal data and 2-tailed Welch t-tests on continuous data, to measure whether groups were different in any way at the beginning of the study.

To assess the direct treatment effects on each primary outcome data we conducted repeated measures ANOVA with arm (experimental/control) as a between-group factor, as well as time point (T1/T2) as within-group factor. We repeated the analyses to test treatment stability, including the scores obtained before vs. 1 month after intervention (T1/T3).

After the intervention (T2) we calculated descriptive statistics on human-chatbot interaction measures as well as the frequency of use (subjective measures and metadata), declared time spent on intervention, and knowledge test score. We also analyzed Pearson correlations between HCI measures."

12a-i) Imputation techniques to deal with attrition / missing values

Imputation techniques to deal with attrition / missing values: Not all participants will use the intervention/comparator as intended and attrition is typically high in ehealth trials. Specify how participants who did not use the application or dropped out from the trial were treated in the statistical analysis (a complete case analysis is strongly discouraged, and simple imputation techniques such as LOCF may also be problematic [4]).

1 2 3 4 5

subitem not at all important ○ ○ ○ ○ ○ essential

Does your paper address subitem 12a-i? *

Copy and paste relevant sections from the manuscript (include quotes in quotation marks "like this" to indicate direct quotes from your manuscript), or elaborate on this item by providing additional information not in the ms, or briefly explain why the item is not applicable/relevant for your study

We didn't use imputation techniques and utilized the standard complete case analysis. Our approach was based on the recommendations by Jakobsen et al. (https://doi.org/10.1186/s12874-017-0442-1) and the results of Jamshidian and Jalal's non-parametric MCAR test (https://doi.org/10.1007/s11336-010-9175-3), which we performed on the dataset consisting of all variables related to our primary hypotheses – the results of this analysis didn't provide any evidence for MAR patterns.

12b) Methods for additional analyses, such as subgroup analyses and adjusted analyses

Does your paper address CONSORT subitem 12b? *

Copy and paste relevant sections from the manuscript (include quotes in quotation marks "like this" to indicate direct quotes from your manuscript), or elaborate on this item by providing additional information not in the ms, or briefly explain why the item is not applicable/relevant for your study

Yes, like this:
"Exploratory analyses
We extended our preregistered analyses after gaining insight into data."

X26) REB/IRB Approval and Ethical Considerations [recommended as subheading under "Methods"] (not a CONSORT item)

X26-i) Comment on ethics committee approval

	1	2	3	4	5	
subitem not at all important	○	○	○	○	○	essential

Does your paper address subitem X26-i?

Copy and paste relevant sections from the manuscript (include quotes in quotation marks "like this" to indicate direct quotes from your manuscript), or elaborate on this item by providing additional information not in the ms, or briefly explain why the item is not applicable/relevant for your study

Yes, like this:
"Ethics and Informed Consent
The study has received approval from the Ethical Review Board at SWPS University of Social Sciences and Humanities (opinion no. 2022-158) and was registered during participant enrollment in OSF Registries (2022)."

x26-ii) Outline informed consent procedures

Outline informed consent procedures e.g., if consent was obtained offline or online (how? Checkbox, etc.?), and what information was provided (see 4a-ii). See [6] for some items to be included in informed consent documents.

	1	2	3	4	5	
subitem not at all important	○	○	○	○	○	essential

Does your paper address subitem X26-ii?

Copy and paste relevant sections from the manuscript (include quotes in quotation marks "like this" to indicate direct quotes from your manuscript), or elaborate on this item by providing additional information not in the ms, or briefly explain why the item is not applicable/relevant for your study

Yes, like this:
"Participants were informed about each intervention, step-by-step procedure, their right to withdraw, and research data confidentiality. All subjects expressed their consent via a

checkbox on a screening survey."

X26-iii) Safety and security procedures

Safety and security procedures, incl. privacy considerations, and any steps taken to reduce the likelihood or detection of harm (e.g., education and training, availability of a hotline)

	1	2	3	4	5	
subitem not at all important	○	○	○	○	○	essential

Does your paper address subitem X26-iii?

Copy and paste relevant sections from the manuscript (include quotes in quotation marks "like this" to indicate direct quotes from your manuscript), or elaborate on this item by providing additional information not in the ms, or briefly explain why the item is not applicable/relevant for your study

We didn't have any special safety and security procedures.

RESULTS

13a) For each group, the numbers of participants who were randomly assigned, received intended treatment, and were analysed for the primary outcome

NPT: The number of care providers or centers performing the intervention in each group and the number of patients treated by each care provider in each center

Does your paper address CONSORT subitem 13a? *

Copy and paste relevant sections from the manuscript (include quotes in quotation marks "like this" to indicate direct quotes from your manuscript), or elaborate on this item by providing additional information not in the ms, or briefly explain why the item is not applicable/relevant for your study

Yes, it is outlined in a participant flow diagram.

13b) For each group, losses and exclusions after randomisation, together with reasons

Does your paper address CONSORT subitem 13b? (NOTE: Preferably, this is shown in a CONSORT flow diagram) *

Copy and paste relevant sections from the manuscript (include quotes in quotation marks "like this" to indicate direct quotes from your manuscript), or elaborate on this item by providing additional information not in the ms, or briefly explain why the item is not applicable/relevant for your study

Yes, it is outlined in a participant flow diagram.

13b-i) Attrition diagram

Strongly recommended: An attrition diagram (e.g., proportion of participants still logging in or using the intervention/comparator in each group plotted over time, similar to a survival curve) or other figures or tables demonstrating usage/dose/engagement.

	1	2	3	4	5	
subitem not at all important	○	○	○	○	○	essential

Does your paper address subitem 13b-i?

Copy and paste relevant sections from the manuscript or cite the figure number if applicable (include quotes in quotation marks "like this" to indicate direct quotes from your manuscript), or elaborate on this item by providing additional information not in the ms, or briefly explain why the item is not applicable/relevant for your study

Yes, we included a participant flow diagram.

14a) Dates defining the periods of recruitment and follow-up

Does your paper address CONSORT subitem 14a? *

Copy and paste relevant sections from the manuscript (include quotes in quotation marks "like this" to indicate direct quotes from your manuscript), or elaborate on this item by providing additional information not in the ms, or briefly explain why the item is not applicable/relevant for your study

Yes:
"Participants were recruited via Facebook and Instagram advertisements from May to June 2022 and screened using a Qualtrics online survey (measurement at T0). "

14a-i) Indicate if critical “secular events” fell into the study period

Indicate if critical “secular events” fell into the study period, e.g., significant changes in Internet resources available or “changes in computer hardware or Internet delivery resources”

	1	2	3	4	5	
subitem not at all important	○	○	○	○	○	essential

Does your paper address subitem 14a-i?

Copy and paste relevant sections from the manuscript (include quotes in quotation marks "like this" to indicate direct quotes from your manuscript), or elaborate on this item by providing additional information not in the ms, or briefly explain why the item is not applicable/relevant for your study

Not relevant, no such secular events fell into the study period.

14b) Why the trial ended or was stopped (early)

Does your paper address CONSORT subitem 14b? *

Copy and paste relevant sections from the manuscript (include quotes in quotation marks "like this" to indicate direct quotes from your manuscript), or elaborate on this item by providing additional information not in the ms, or briefly explain why the item is not applicable/relevant for your study

Not relevant, the trial didn't conclude early.

15) A table showing baseline demographic and clinical characteristics for each group

NPT: When applicable, a description of care providers (case volume, qualification, expertise, etc.) and centers (volume) in each group

Does your paper address CONSORT subitem 15? *

Copy and paste relevant sections from the manuscript (include quotes in quotation marks "like this" to indicate direct quotes from your manuscript), or elaborate on this item by providing additional information not in the ms, or briefly explain why the item is not applicable/relevant for your study

Yes, we included the demographic table.

15-i) Report demographics associated with digital divide issues

In ehealth trials it is particularly important to report demographics associated with digital divide issues, such as age, education, gender, social-economic status, computer/Internet/ehealth literacy of the participants, if known.

	1	2	3	4	5	
subitem not at all important	○	○	○	○	○	essential

Does your paper address subitem 15-i? *

Copy and paste relevant sections from the manuscript (include quotes in quotation marks "like this" to indicate direct quotes from your manuscript), or elaborate on this item by providing additional information not in the ms, or briefly explain why the item is not applicable/relevant for your study

Partly. We included basic variables such as age, education, gender, but the social-economic status was not assessed.

16) For each group, number of participants (denominator) included in each analysis and whether the analysis was by original assigned groups

16-i) Report multiple “denominators” and provide definitions

Report multiple “denominators” and provide definitions: Report N’s (and effect sizes) “across a range of study participation [and use] thresholds” [1], e.g., N exposed, N consented, N used more than x times, N used more than y weeks, N participants “used” the intervention/comparator at specific pre-defined time points of interest (in absolute and relative numbers per group). Always clearly define “use” of the intervention.

1 2 3 4 5

subitem not at all important ○ ○ ○ ○ ○ essential

Does your paper address subitem 16-i? *

Copy and paste relevant sections from the manuscript (include quotes in quotation marks "like this" to indicate direct quotes from your manuscript), or elaborate on this item by providing additional information not in the ms, or briefly explain why the item is not applicable/relevant for your study

Partly. N's are presented in the participant flow diagram.

16-ii) Primary analysis should be intent-to-treat

Primary analysis should be intent-to-treat, secondary analyses could include comparing only “users”, with the appropriate caveats that this is no longer a randomized sample (see 18-i).

	1	2	3	4	5	
subitem not at all important	○	○	○	○	○	essential

Does your paper address subitem 16-ii?

Copy and paste relevant sections from the manuscript (include quotes in quotation marks "like this" to indicate direct quotes from your manuscript), or elaborate on this item by providing additional information not in the ms, or briefly explain why the item is not applicable/relevant for your study

Yes, we used intention-to-treat analysis.

17a) For each primary and secondary outcome, results for each group, and the estimated effect size and its precision (such as 95% confidence interval)

Does your paper address CONSORT subitem 17a? *

Copy and paste relevant sections from the manuscript (include quotes in quotation marks "like this" to indicate direct quotes from your manuscript), or elaborate on this item by providing additional information not in the ms, or briefly explain why the item is not applicable/relevant for your study

not applicable/relevant for your study

Yes, we used effect sizes estimates with 95% CI.

17a-i) Presentation of process outcomes such as metrics of use and intensity of use

In addition to primary/secondary (clinical) outcomes, the presentation of process outcomes such as metrics of use and intensity of use (dose, exposure) and their operational definitions is critical. This does not only refer to metrics of attrition (13-b) (often a binary variable), but also to more continuous exposure metrics such as “average session length”. These must be accompanied by a technical description how a metric like a “session” is defined (e.g., timeout after idle time) [1] (report under item 6a).

	1	2	3	4	5	
subitem not at all important	○	○	○	○	○	essential

Does your paper address subitem 17a-i?

Copy and paste relevant sections from the manuscript (include quotes in quotation marks "like this" to indicate direct quotes from your manuscript), or elaborate on this item by providing additional information not in the ms, or briefly explain why the item is not applicable/relevant for your study

We only collected and reported basic metrics of use, like this:
"Groups were similar in terms of the frequency of use they reported after the intervention (t63=1.44, P=.15, d=0.36, 95% CI −0.13 to 0.84) and scores on the knowledge test (t57=0.02, P=.99, d=0.01, 95% CI −0.51 to 0.52). However, the analysis of time spent on the intervention via regular commitment checks suggested that the control group (mean 117.57 minutes, SD 72.40) actually spent more time reading the book than the chatbot group (mean 79.44 minutes, SD 42.96) spent on the interaction with Fido, t59=2.75, P=.008, d=0.64 (95% CI 0.17-1.11)."

17b) For binary outcomes, presentation of both absolute and relative effect sizes is recommended

Does your paper address CONSORT subitem 17b? *

Copy and paste relevant sections from the manuscript (include quotes in quotation marks "like this" to indicate direct quotes from your manuscript), or elaborate on this item by providing additional information not in the ms, or briefly explain why the item is not applicable/relevant for your study

No binary outcomes were used in the study.

18) Results of any other analyses performed, including subgroup analyses and adjusted analyses, distinguishing pre-specified from exploratory

Does your paper address CONSORT subitem 18? *

Copy and paste relevant sections from the manuscript (include quotes in quotation marks "like this" to indicate direct quotes from your manuscript), or elaborate on this item by providing additional information not in the ms, or briefly explain why the item is not applicable/relevant for your study

Yes, the exploratory analyses are distinguished from the preregistered.

18-i) Subgroup analysis of comparing only users

A subgroup analysis of comparing only users is not uncommon in ehealth trials, but if done, it must be stressed that this is a self-selected sample and no longer an unbiased sample from a randomized trial (see 16-iii).

	1	2	3	4	5	
subitem not at all important	○	○	○	○	○	essential

Does your paper address subitem 18-i?

Copy and paste relevant sections from the manuscript (include quotes in quotation marks "like this" to indicate direct quotes from your manuscript), or elaborate on this item by providing additional information not in the ms, or briefly explain why the item is not applicable/relevant for your study

Twoja odpowiedź

19) All important harms or unintended effects in each group
(for specific guidance see CONSORT for harms)

Does your paper address CONSORT subitem 19? *

Copy and paste relevant sections from the manuscript (include quotes in quotation marks "like this" to indicate direct quotes from your manuscript), or elaborate on this item by providing additional information not in the ms, or briefly explain why the item is not applicable/relevant for your study

Yes, like this:
"The most common response about the negative aspects of using Fido was that there were many failures to recognise the user's intent, which resulted in the chatbot telling the user that it does not know what they are talking about."

19-i) Include privacy breaches, technical problems

Include privacy breaches, technical problems. This does not only include physical "harm" to participants, but also incidents such as perceived or real privacy breaches [1], technical problems, and other unexpected/unintended incidents. "Unintended effects" also includes unintended positive effects [2].

	1	2	3	4	5	
subitem not at all important	○	○	○	○	○	essential

Does your paper address subitem 19-i?

Copy and paste relevant sections from the manuscript (include quotes in quotation marks "like this" to indicate direct quotes from your manuscript), or elaborate on this item by providing additional information not in the ms, or briefly explain why the item is not applicable/relevant for your study

Yes, like this:
"Due to the Messenger chatbot policies [24], the research team members were hypothetically able to access individual participant messages from the chatbot conversations. This limitation is due to the fact that, formally, Messenger chatbots are an interface to send messages to Facebook pages. Participants from the experimental condition were informed of this potential privacy breach via a consent form explicitly presented in the chatbot and Meta's Data Policy [25]."

19-ii) Include qualitative feedback from participants or observations from staff/researchers

Include qualitative feedback from participants or observations from staff/researchers, if available, on strengths and shortcomings of the application, especially if they point to unintended/unexpected effects or uses. This includes (if available) reasons for why people did or did not use the application as intended by the developers.

1	2	3	4	5

subitem not at all important ○ ○ ○ ○ ○ essential

Does your paper address subitem 19-ii?

Copy and paste relevant sections from the manuscript (include quotes in quotation marks "like this" to indicate direct quotes from your manuscript), or elaborate on this item by providing additional information not in the ms, or briefly explain why the item is not applicable/relevant for your study

Yes, like this:
"The team also collected informal, qualitative data through individual in-depth interviews and an open-ended survey question after the intervention (T2). Volunteers (N=16) from the chatbot group answered questions about their perceived advantages and pitfalls of Fido, as well as other unintended effects which had not been assessed by the previously administered questionnaires. The most common response about the negative aspects of using Fido was that there were many failures to recognise the user's intent, which resulted in the chatbot telling the user that it does not know what they are talking about. From the positive side effects, the most prominent was that using Fido encouraged the user to make a decision about starting the traditional therapy. "

DISCUSSION

22) Interpretation consistent with results, balancing benefits and harms, and considering other relevant evidence

NPT: In addition, take into account the choice of the comparator, lack of or partial blinding, and unequal expertise of care providers or centers in each group

22-i) Restate study questions and summarize the answers suggested by the data, starting with primary outcomes and process outcomes (use)

Restate study questions and summarize the answers suggested by the data, starting with primary outcomes and process outcomes (use).

	1	2	3	4	5	
subitem not at all important	○	○	○	○	○	essential

Does your paper address subitem 22-i? *

Copy and paste relevant sections from the manuscript (include quotes in quotation marks "like this" to indicate direct quotes from your manuscript), or elaborate on this item by providing additional information not in the ms, or briefly explain why the item is not applicable/relevant for your study

Yes, these aspects are discussed.

22-ii) Highlight unanswered new questions, suggest future research

Highlight unanswered new questions, suggest future research.

	1	2	3	4	5	
subitem not at all important	○	○	○	○	○	essential

Does your paper address subitem 22-ii?

Copy and paste relevant sections from the manuscript (include quotes in quotation marks "like this" to indicate direct quotes from your manuscript), or elaborate on this item by providing additional information not in the ms, or briefly explain why the item is not applicable/relevant for your study

Yes, these are discussed.

20) Trial limitations, addressing sources of potential bias, imprecision, and, if relevant, multiplicity of analyses

20-i) Typical limitations in ehealth trials

Typical limitations in ehealth trials: Participants in ehealth trials are rarely blinded. Ehealth trials often look at a multiplicity of outcomes, increasing risk for a Type I error. Discuss biases due to non-use of the intervention/usability issues, biases through informed consent procedures, unexpected events.

	1	2	3	4	5	
subitem not at all important	○	○	○	○	○	essential

Does your paper address subitem 20-i? *

Copy and paste relevant sections from the manuscript (include quotes in quotation marks "like this" to indicate direct quotes from your manuscript), or elaborate on this item by providing additional information not in the ms, or briefly explain why the item is not applicable/relevant for your study

Yes, many of these aspects are discussed.

21) Generalisability (external validity, applicability) of the trial findings

NPT: External validity of the trial findings according to the intervention, comparators, patients, and care providers or centers involved in the trial

21-i) Generalizability to other populations

Generalizability to other populations: In particular, discuss generalizability to a general Internet population, outside of a RCT setting, and general patient population, including applicability of the study results for other organizations

	1	2	3	4	5	
subitem not at all important	○	○	○	○	○	essential

Does your paper address subitem 21-i?

Copy and paste relevant sections from the manuscript (include quotes in quotation marks "like this" to indicate direct quotes from your manuscript), or elaborate on this item by providing additional information not in the ms, or briefly explain why the item is not applicable/relevant for your study

Twoja odpowiedź

21-ii) Discuss if there were elements in the RCT that would be different in a routine application setting

Discuss if there were elements in the RCT that would be different in a routine application setting (e.g., prompts/reminders, more human involvement, training sessions or other co-interventions) and what impact the omission of these elements could have on use, adoption, or outcomes if the intervention is applied outside of a RCT setting.

	1	2	3	4	5	
subitem not at all important	○	○	○	○	○	essential

Does your paper address subitem 21-ii?

Copy and paste relevant sections from the manuscript (include quotes in quotation marks "like this" to indicate direct quotes from your manuscript), or elaborate on this item by providing additional information not in the ms, or briefly explain why the item is not applicable/relevant for your study

Yes, we discuss the standard application setting (outside of this RCT).

OTHER INFORMATION

23) Registration number and name of trial registry

Does your paper address CONSORT subitem 23? *

Copy and paste relevant sections from the manuscript (include quotes in quotation marks "like this" to indicate direct quotes from your manuscript), or elaborate on this item by providing additional information not in the ms, or briefly explain why the item is not applicable/relevant for your study

ClinicalTrials.gov Registry: NCT05762939 (retrospective)
Open Science Foundation Registry: 2cqt3 (prospective)

24) Where the full trial protocol can be accessed, if available

Does your paper address CONSORT subitem 24? *

Cite a Multimedia Appendix, other reference, or copy and paste relevant sections from the manuscript (include quotes in quotation marks "like this" to indicate direct quotes from your manuscript), or elaborate on this item by providing additional information not in the ms, or briefly explain why the item is not applicable/relevant for your study

We didn't create a full study protocol, but the OSF preregistration (https://osf.io/2cqt3) should be treated as its minimal version.

25) Sources of funding and other support (such as supply of drugs), role of funders

Does your paper address CONSORT subitem 25? *

Copy and paste relevant sections from the manuscript (include quotes in quotation marks "like this" to indicate direct quotes from your manuscript), or elaborate on this item by providing additional information not in the ms, or briefly explain why the item is not applicable/relevant for your study

Yes, like this:
"This work was supported by Ministry of Science and Higher Education grant 012/RID/2018/19."

X27) Conflicts of Interest (not a CONSORT item)

X27-i) State the relation of the study team towards the system being evaluated

In addition to the usual declaration of interests (financial or otherwise), also state the relation of the study team towards the system being evaluated, i.e., state if the authors/evaluators are distinct from or identical with the developers/sponsors of the intervention.

	1	2	3	4	5	
subitem not at all important	○	○	○	○	○	essential

Does your paper address subitem X27-i?

Copy and paste relevant sections from the manuscript (include quotes in quotation marks "like this" to indicate direct quotes from your manuscript), or elaborate on this item by providing additional information not in the ms, or briefly explain why the item is not applicable/relevant for your study

Yes, like this:
"All authors are actively involved in the development of Fido at a pre-release stage yet declare no financial benefits from this project other than from a publicly funded grant mentioned above. "

About the CONSORT EHEALTH checklist

As a result of using this checklist, did you make changes in your manuscript? *

◉ yes, major changes

- [illegible]
- ( ) yes, minor changes
- ( ) no

What were the most important changes you made as a result of using this checklist?

Twoja odpowiedź

How much time did you spend on going through the checklist INCLUDING making changes in your manuscript *

Difficult to estimate, but probably around 3-7 hours.

As a result of using this checklist, do you think your manuscript has improved? *

- (•) yes
- ( ) no
- ( ) Inne:

Would you like to become involved in the CONSORT EHEALTH group?

This would involve for example becoming involved in participating in a workshop and writing an "Explanation and Elaboration" document

- ( ) yes
- (•) no
- ( ) Inne:

Odznacz

Any other comments or questions on CONSORT EHEALTH

Twoja odpowiedź

[illegible]

STOP - Save this form as PDF before you click submit
To generate a record that you filled in this form, we recommend to generate a PDF of this page (on a Mac, simply select "print" and then select "print as PDF") before you submit it.

When you submit your (revised) paper to JMIR, please upload the PDF as supplementary file.

Don't worry if some text in the textboxes is cut off, as we still have the complete information in our database. Thank you!

Final step: Click submit !
Click submit so we have your answers in our database!

Prześlij

Wyczyść formularz

Nigdy nie podawaj w Formularzach Google swoich haseł.

Ten formularz został utworzony poza Twoją domeną. Zgłoś nadużycie - Warunki korzystania z usługi - Ochrona danych osobowych

Formularze Google
